# Single-photon superradiance in individual caesium lead halide quantum dots

**DOI:** 10.1038/s41586-023-07001-8

**Published:** 2024-01-31

**Authors:** Chenglian Zhu, Simon C. Boehme, Leon G. Feld, Anastasiia Moskalenko, Dmitry N. Dirin, Rainer F. Mahrt, Thilo Stöferle, Maryna I. Bodnarchuk, Alexander L. Efros, Peter C. Sercel, Maksym V. Kovalenko, Gabriele Rainò

**Affiliations:** 1 Department of Chemistry and Applied Biosciences, Institute of Inorganic Chemistry, ETH Zürich, Zürich, Switzerland; 2 Laboratory for Thin Films and Photovoltaics, Empa—Swiss Federal Laboratories for Materials Science and Technology, Dübendorf, Switzerland; 3 IBM Research Europe—Zurich, Rüschlikon, Switzerland; 4 Center for Computational Materials Science, US Naval Research Laboratory, Washington DC, USA; 5 Center for Hybrid Organic Inorganic Semiconductors for Energy, Golden, CO USA

**Keywords:** Quantum dots, Single photons and quantum effects

## Abstract

The brightness of an emitter is ultimately described by Fermi’s golden rule, with a radiative rate proportional to its oscillator strength times the local density of photonic states. As the oscillator strength is an intrinsic material property, the quest for ever brighter emission has relied on the local density of photonic states engineering, using dielectric or plasmonic resonators^1,2^. By contrast, a much less explored avenue is to boost the oscillator strength, and hence the emission rate, using a collective behaviour termed superradiance. Recently, it was proposed^3^ that the latter can be realized using the giant oscillator-strength transitions of a weakly confined exciton in a quantum well when its coherent motion extends over many unit cells. Here we demonstrate single-photon superradiance in perovskite quantum dots with a sub-100 picosecond radiative decay time, almost as short as the reported exciton coherence time^4^. The characteristic dependence of radiative rates on the size, composition and temperature of the quantum dot suggests the formation of giant transition dipoles, as confirmed by effective-mass calculations. The results aid in the development of ultrabright, coherent quantum light sources and attest that quantum effects, for example, single-photon emission, persist in nanoparticles ten times larger than the exciton Bohr radius.

## Abstract (web summary)

Excitonic single-photon superradiance is reported in individual perovskite quantum dots with a sub-100 ps radiative decay time, almost as short as the reported exciton coherence time.

## Main

Research on CsPbX_3_ (X = Cl, Br, I) lead-halide perovskite quantum dots (QDs) has been intense since their first colloidal synthesis in 2015^5^. The CsPbX_3_ QDs are appealing because of facile yet precise control over QD size, shape and composition, as well as spectrally narrow photoluminescence with near-unity quantum yield, facilitated by a defect-tolerant electronic structure. Perovskite-QD-based optoelectronic devices with unprecedented performance include LEDs with external quantum efficiencies above 20% (refs. ^6,7^), lasers with wide spectral tunability^8,9^ and photodetectors with record-high responsivity^10^. Parallel to the development of classical light-emitting devices, CsPbX_3_ QDs are also explored as sources of quantum light, capable of delivering either single photons^4,11–13^ or bunched (temporally correlated) multi-photon bundles^14^. However, despite the widespread use of CsPbX_3_ QDs in photonic applications, exciton recombination in these nanomaterials remains poorly understood. For instance, the strong temperature dependence of the exciton radiative rate, with an acceleration by more than two orders of magnitudes on cooling from room temperature to cryogenic temperature, remains unanswered.

Emitters with high radiative rates are highly sought after in several classical and quantum light applications^15,16^, as a rapid radiative decay can (1) boost the material gain in laser devices; (2) increase the brightness—that is, the number of generated photons per unit time—of light-emitting devices, including single-photon sources; and (3) enable the realization of coherent single-photon sources delivering indistinguishable single photons^4,17^. In general, photon emission in semiconductors results from spontaneous radiative recombination of excitons induced by zero-point fluctuations of the vacuum mode of the electromagnetic field. This leads to an excited state with a finite lifetime, described by Fermi’s golden rule, which states that the transition rate is proportional to the product of the modulus squared of the matrix element describing the optical transition and the local density of photonic states (LDOS) at the emitting energy.

The efforts to increase radiative decay rates have mostly focused on exploiting plasmonic or dielectric microcavities, thus enhancing the LDOS using the so-called Purcell effect^1^. In a recent effort^2^ exploiting InGaAs QDs in one-dimensional optical microcavities, radiative lifetimes down to about 48 ps were obtained with more than 97.5% indistinguishability.

Alternatively, strongly enhanced emission rates can be accomplished by collective behaviour in a process called superradiance, proposed by Dicke in 1954 for a dense atom cloud^18^ and recently reported in semiconductor systems^14,19,20^. When the emitters in the medium are strongly coupled using the vacuum modes of the electromagnetic field, coherent excitation results in an ensemble of phase-locked emitters that behaves as a giant dipole with an oscillator strength scaling with *N*, the number of correlated emitters^18,21^. This giant dipole then radiates spontaneously with a higher rate (∝*N*) and much stronger peak intensity (∝*N*^2^) than an ensemble of incoherent emitters. Superradiance can also emerge when only one photon is stored in an ensemble of coupled emitters^22^. This so-called single-photon superradiance (SPS) has become a subject of recent interest^23^ and offers potential applications for quantum control of spontaneous emission and ultrafast readout.

**Fig. 1:**
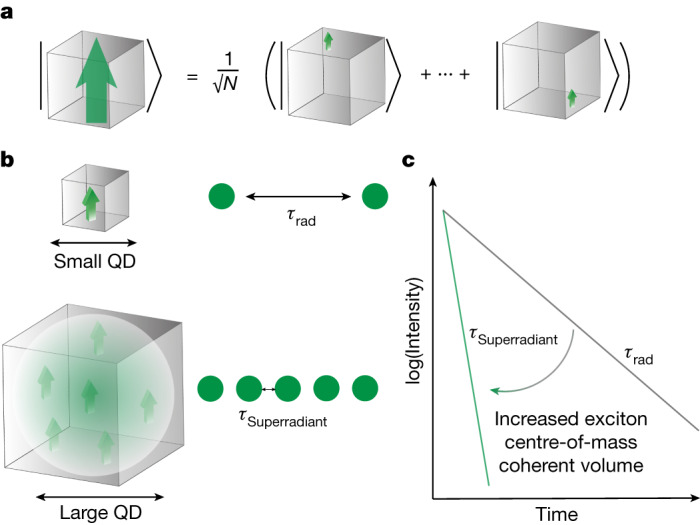
Schematic of the excitonic SPS in solid-state quantum emitters. **a**, SPS originates from the coherent coupling of individual excitations (small arrows), generating a giant dipole (large arrow); ideally, the coherent coupling spans a region much larger than the exciton Bohr diameter. **b**, The formation of giant dipoles, while geometrically clamped for small QDs, can be achieved in large QDs and leads to an accelerated radiative decay (with a lifetime *τ*_Superradiant_) because of the increased exciton centre-of-mass coherent volume. **c**, Schematic showing the photoluminescence decay without superradiance (with lifetime *τ*_rad_, characteristic of QDs in the strong quantum confinement; grey line) and with superradiance (with a reduced lifetime *τ*_Superradiant_, reachable for QDs in the very weak confinement regime; green line).

SPS can even occur in a single semiconductor QD, referred to as excitonic SPS, as shown in Fig. 1: in QDs larger than the exciton Bohr diameter, superradiant states can be formed because of effective exciton wavefunction delocalization over a region much larger than the Bohr diameter. Consequently, all the unit cells in the coherence volume cooperatively respond to an excitation, behaving like a giant transition dipole with enhanced oscillator strength. The emission thereby features an accelerated radiative decay with the maximum rate enhancement scaling with the number of unit cells involved in the exciton coherence volume^24,25^. It is also important to recognize that there is a formal correspondence between the collective emission of an exciton in the intermediate or weak confinement regimes, in which the motion of the electron and the hole are correlated, and the N-atom single-photon Dicke state^3^ (Supplementary Information section II). In an experimental realization of SPS, the exciton delocalization could be geometrically limited by the spatial extent of the QD, by the different Bohr radii in different compounds, and eventually hampered by the exciton–phonon coupling that prevents efficient exciton wavefunction delocalization on increasing the crystal temperature. So far, accelerated radiative decays have been demonstrated in CuCl microcrystallites^25,26^, CdS microcrystallites^27^, CdSe nanoplatelets^28,29^ and molecular J-aggregates^30^. However, single-photon emission through excitonic SPS was only recently achieved in weakly confined GaAs QDs, defined by random local thickness variations within a quantum well^3^, reaching a radiative lifetime of about 90 ps. Using the envelope function formalism, the latter work established the first connection between the photophysics of an exciton in the weakly confined regime and Dicke’s superradiance and provided a theoretical estimate of the size-dependent transition strength.

In this work, we show that the light emission process in CsPbX_3_ (X = Br, Br/Cl) perovskite QDs at low temperature is governed by excitonic SPS, evidenced by the characteristic size-, composition-, and temperature-dependent radiative lifetime. We obtain sub-100 ps radiative lifetimes in large QDs with edge lengths of more than three times the Bohr diameter. Despite their weak confinement, these large QDs are efficient emitters of single photons, as attested by second-order correlation measurements. Our findings provide an opportunity for an order of magnitude brighter quantum light sources based on inexpensive, solution-processable and wavelength-tunable all-inorganic perovskite QDs.

## Size-dependent exciton lifetime

**Fig. 2:**
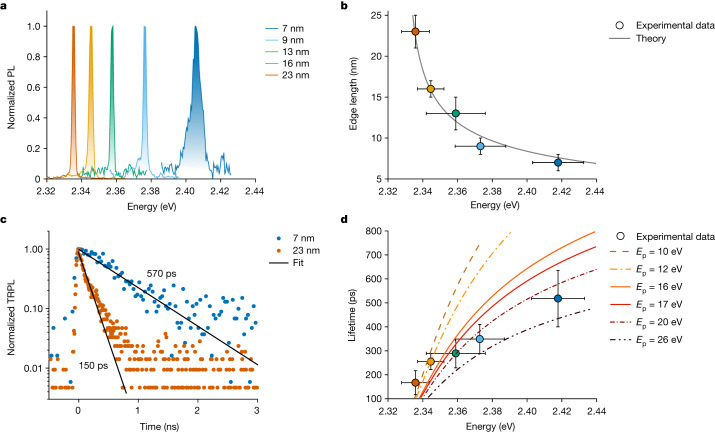
Size-dependent exciton lifetime at 4 K. **a**, Representative photoluminescence (PL) spectra of single CsPbBr_3_ perovskite QDs with different sizes. **b**, Size-dependent mean exciton photoluminescence band-edge emission at 4 K; vertical and horizontal error bars represent the standard deviation of the QD edge length and exciton energy, respectively. The grey solid line is a sizing curve obtained by using an effective-mass model for intermediate confinement. **c**, TRPL decay of two single QDs in the weak- to intermediate-confinement regime (QD size of 7 nm; blue dots) and weak confinement regime (QD size of 23 nm; vermillion dots), respectively. The solid grey lines indicate single-exponential decays with inferred time constants (defined as 1/e intensity decrease) of 570 ps and 150 ps, for the 7 nm and 23 nm QD, respectively. **d**, Size-dependent mean exciton lifetime as a function of the exciton energy, acquired at 4 K (coloured circles); vertical and horizontal error bars represent the standard deviation of lifetime and exciton energy, respectively. The lines show the result of theoretical calculations, performed at 0 K and assuming various values for the Kane energy (*E*_p_) shown as dashed and solid lines as indicated in the legend. More details concerning the theoretical model are reported in Supplementary Information.

**Extended Data Fig. 1:**
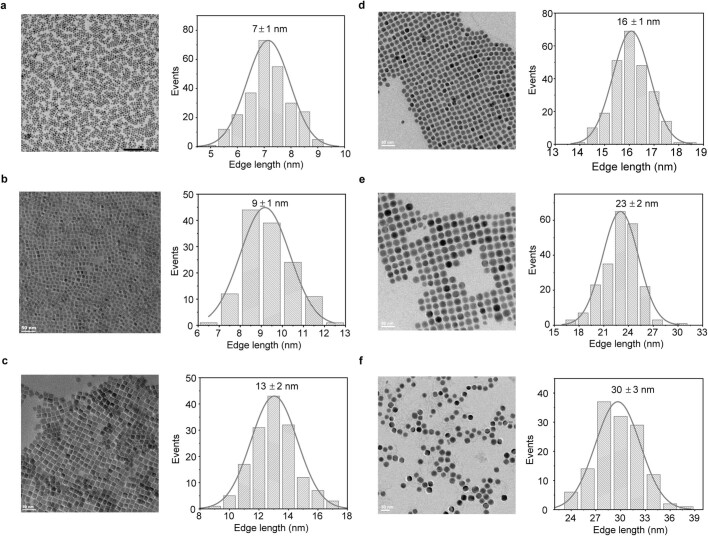
Size characterization of the studied samples. **a**–**f**, The TEM images and corresponding size distribution of the samples with mean edge lengths of ca. 7 nm, 9 nm, 13 nm, 16 nm, 23 nm, and 30 nm, respectively.

As shown in Fig. 1, excitonic SPS can be achieved by exploring QDs with increasing sizes. SPS should manifest itself in decreasing radiative lifetimes with increasing QD sizes, as more unit cells can contribute to the collective emission and, hence, increase the exciton coherent volume. We, therefore, recorded the photoluminescence of more than 200 single CsPbBr_3_ QDs at cryogenic temperature (4 K), obtained from five different QD batches with mean QD sizes ranging from 7 nm to 23 nm (Extended Data Fig. 1). Considering the Bohr diameter of approximately 7 nm in CsPbBr_3_ (ref. ^5^), the probed QD size range extends from an intermediate to a very weak quantum confinement regime. Figure 2a shows representative photoluminescence spectra from single CsPbBr_3_ QDs of the above-mentioned samples exhibiting a confinement-induced increase in exciton energy with decreasing QD size. Figure 2b shows the corresponding mean value and standard deviation of the single-QD exciton energy for each batch, showing a very good agreement with the size-dependent energies predicted by a variational effective-mass/intermediate-confinement model that includes band non-parabolicity corrections (details in Supplementary Information section II). In the intermediate-confinement regime, the correlated electron–hole motion of the exciton in conjunction with the overall exciton centre-of-mass delocalization enhances the radiative^11,31^ and non-radiative decay rates^32^ beyond those expected within a simple strong confinement approximation. In Supplementary Information section II and Supplementary Fig. 3, we show that these effects are captured by the variational method, reproducing the effects of correlation found using all-order many-body approaches within the intermediate-confinement size regime calculated for the same parameter set^31^.

**Extended Data Fig. 2:**
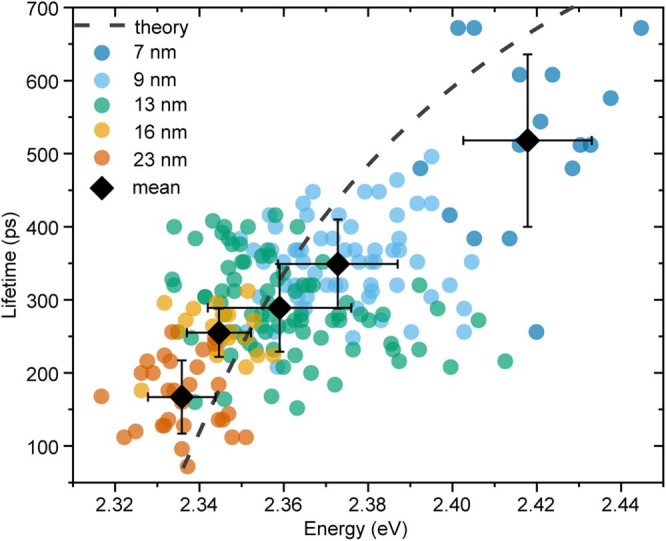
Size-dependent exciton lifetime. Characteristic size-dependent exciton lifetime as a function of the exciton energy, for various single QDs (circles) and their mean value per QD batch (diamonds), respectively. Error bars are standard deviations. Theoretical curve (dashed line) is from calculation with ${E}_{p}=17\,eV$.

For each of the probed single QDs, a time-resolved photoluminescence (TRPL) trace was recorded at the exciton emission peak energy. Figure 2c shows that the excited-state lifetime (defined using a 1/e intensity decrease) of a representative 23 nm QD (150 ps) is considerably shorter than that of a representative 7 nm QD (570 ps). For each of the QD batches, Fig. 2d shows the mean single-QD lifetime as a function of the mean exciton energy. A monotonous decrease in the mean single-QD lifetime from 540 ± 100 ps to 170 ± 50 ps is observed for mean QD sizes from 7 nm to 23 nm. The error bars (standard deviation) represent the QD-to-QD variations within the sample (see Extended Data Fig. 2 for the lifetimes of all studied individual QDs). Contrary to II–VI and III–V compounds, in which the radiative process is largely dependent on the dark-bright exciton interaction^33^, the observed photoluminescence decay in this work reflects a bright exciton recombination process because of the highly suppressed relaxation to the dark state at cryogenic temperatures and at zero magnetic field^34^.

The speed-up of the decay with increasing CsPbBr_3_ QD size at cryogenic temperature is also opposite to the respective trend at room temperature^35^, at which thermal mixing of band-edge states with higher-energy exciton states whose radiative decay is parity-forbidden (for example, transitions from *s*-like confined states in one band to *p*-like confined states in the other band) causes slower radiative decay in larger CsPbBr_3_ QDs^35^.

**Extended Data Fig. 3:**
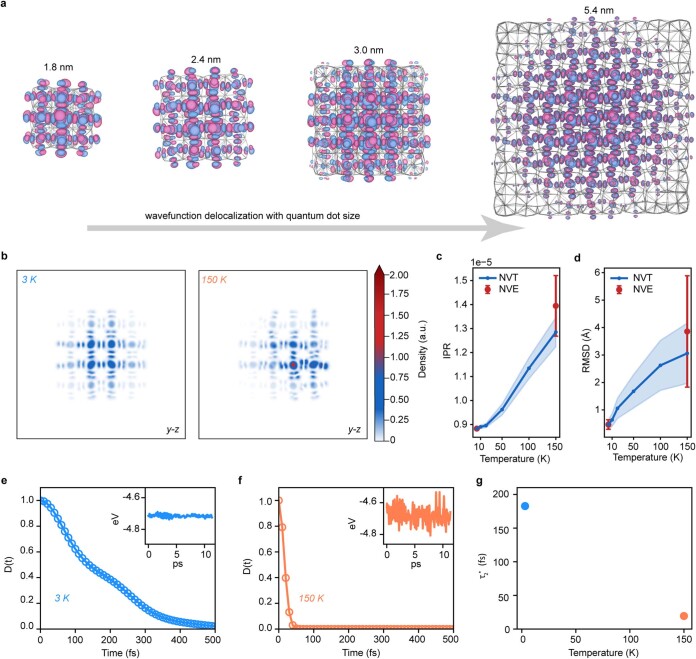
Atomistic insight into electronic wavefunction delocalization and dephasing in CsPbBr_3_ quantum dots *via ab initio* molecular dynamics at the DFT level of theory. **a**, The HOMO wavefunction delocalizes across the quantum dot and increases in size as the quantum dot diameter increases from 1.8 nm to 5.4 nm. **b**, Representative snapshot of the HOMO density at 3 K (left) and 150 K (right) projected onto the y-z plane. **c**, Time-averaged inverse participation ratio (IPR; degree of localization) of the HOMO density obtained at various temperatures, revealing thermally induced localization of the HOMO density. Simulations performed in the canonical ensemble (NVT, blue) and the microcanonical ensemble (NVE, red) return the similar localization. Error bars indicate 95% confidence intervals. **d**, Temperature-dependence of the time-averaged root-mean-squared displacement of the HOMO density center from the QD center, showing thermally induced dynamic spatial fluctuations. Error bars indicate 95% confidence intervals. **e**–**g**, Phonon-induced electronic dephasing in CsPbBr_3_ QDs: the dephasing function D(t) related to the HOMO at (e) 3 K and (f) 150 K, respectively, is obtained from the temporal fluctuations of the HOMO energy (see insets). **g**, The extracted pure-dephasing time τ_2_^*^ strongly decreases with increasing temperature.

Our observation of accelerated radiative decay at cryogenic temperatures can be explained by the enhanced oscillator strength in larger QDs because of exciton wavefunction delocalization. To test this hypothesis, we developed an effective-mass model that captures the exciton wavefunction delocalization and the resulting enhancement of the oscillator strength for larger QDs (Extended Data Fig. 3a and Supplementary Information section III). A remaining uncertainty is the magnitude of the Kane energy, *E*_p_, which is not a directly measurable parameter. Its value has been estimated from the reduced effective mass of the exciton^36^, yielding estimates of the Kane energy between 16 eV and 28 eV (refs. ^37,38^), or from the hole Landé *g*-factor^39,40^, yielding estimates between 10 eV (for a spin-orbit split-off parameter *Δ* = 1.5 eV; ref. ^37^) and 16 eV (for *Δ* = 0.8 eV; ref. ^31^) (see Supplementary Information section IIB,C for details). To account for the presently large uncertainty in the value of the Kane energy, we calculated the size-dependent radiative lifetime for a range of *E*_p_ values (Fig. 2d). Regardless of the value of *E*_p_, the exciton lifetime consistently reduces with increasing QD size, qualitatively capturing the experimental trend in single-QD lifetimes^3,26,31^. The scenarios with *E*_p_ between 16 eV and 20 eV yield the best overall agreement with the experimental results across the entire size range. Overall, the shortened lifetime with increasing QD size is consistent with exciton wavefunction delocalization in larger QDs, a typical feature of the superradiant excitonic system (for details, see Supplementary Information section IIB,C).

## Temperature-dependent exciton lifetime

**Fig. 3:**
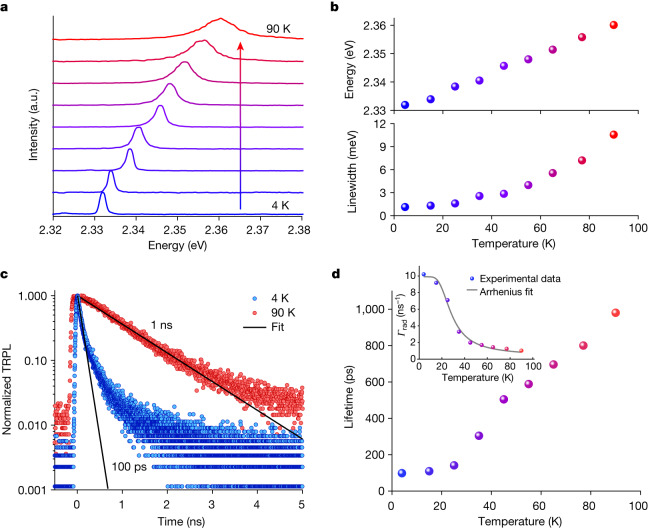
Temperature-dependent exciton lifetime. **a**, Photoluminescence spectra from a single 23-nm CsPbBr_3_ QD as a function of temperature. **b**, Linewidth and exciton peak energy as a function of temperature, extracted from the spectra in **a** by performing single-Lorentzian fitting. With increasing temperature, the exciton photoluminescence emission energy blue shifts and the photoluminescence linewidth increases, as a result of exciton–phonon coupling. **c**, TRPL decay at 4 K (blue markers) and 90 K (red markers). Solid black lines indicate single-exponential decays with time constants of 100 ps at 4 K and 1  ns at 90 K. **d**, Lifetime as a function of temperature (colour coding identical to that in **b**). Inset: the corresponding radiative decay rate (*Γ*_rad_) as a function of temperature fitted by an Arrhenius function (grey solid line). a.u., arbitrary units.

Another key tuning parameter with a potentially profound effect on the superradiant decay is the base crystal temperature. To this aim, we studied the temperature-dependent steady-state and time-resolved photoluminescence properties of a single CsPbBr_3_ QD (about 23 nm). Figure 3a shows the photoluminescence spectrum of a single QD from 4 K up to 90 K, exhibiting a continuous blue shift and photoluminescence line broadening with increasing temperature because of lattice expansion and exciton–phonon coupling, respectively. Exciton energies and linewidth (full-width at half-maximum (FWHM)), determined by Lorentzian fitting, are shown in Fig. 3b. The FWHM increases from an instrument-limited value of 1.2 meV at 4 K to 10.5 meV at 90 K as a result of the strong exciton–phonon coupling in the relatively soft perovskite compounds^41^.

**Extended Data Fig. 4:**
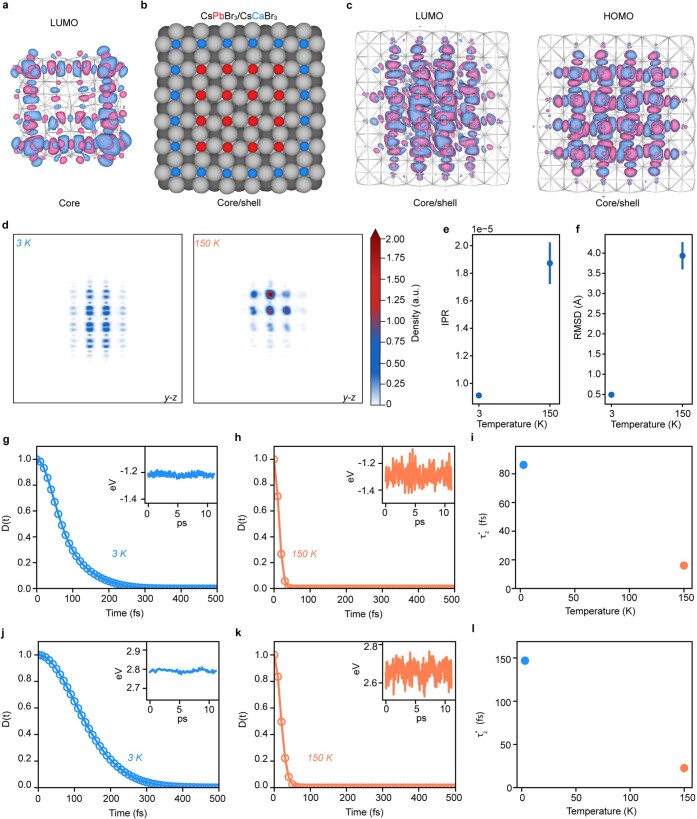
Atomistic insight into electronic wavefunction delocalization in core/shell CsPbBr_3_/CsCaBr_3_ quantum dots *via ab initio* molecular dynamics at the DFT level of theory. **a**, The LUMO density localizes on the surface (edges and corners) in models without surface passivation. **b**, Cross-section of a core/shell CsPbBr_3_/CsCaBr_3_ model revealing the 2.4 nm CsPbBr_3_ core with a single layer of CsCaPb_3_ shell. **c**, LUMO (left) and HOMO (right) wavefunctions of the core/shell model. Both states are fully delocalized across the core due to electronic passivation by the shell. **d**, Representative snapshot of the LUMO density at 3 K (left) and 150 K (right) projected onto the y-z plane. **e**, Time-averaged inverse participation ratio (IPR; degree of localization) of the LUMO density at 3 and 150 K, revealing thermally induced localization. Error bars indicate 95% confidence intervals. **f**, Time-averaged root-mean-squared displacement (RMSD) of the LUMO density center from the QD center at 3 and 150 K, showing thermally induced dynamic spatial fluctuations. Error bars indicate 95% confidence intervals. **g**–**i**, Phonon-induced LUMO dephasing in CsPbBr_3_ QDs: the dephasing function D(t) related to the LUMO at (g) 3 K and (h) 150 K, respectively, is obtained from the temporal fluctuations of the LUMO energy (see insets). **i**, The extracted pure-dephasing time τ_2_^*^ strongly decreases with increasing temperature. **j**–**l**, Phonon-induced band gap dephasing in CsPbBr_3_ QDs, analyzed analogously to the LUMO results in (g)-(i).

Figure 3c shows the TRPL decay traces of a single QD at 4 K and 90 K, with corresponding lifetimes of 100 ps and 1 ns, respectively. As shown in Fig. 3d, the lifetime consistently increases from 100 ps at 4 K to 1 ns at 90 K. This temperature-induced deceleration of the radiative decay in a perovskite QD is in contrast to the respective acceleration of the radiative decay generally observed in traditional (non-perovskite) QDs. Although the latter speed-up originates from a thermal mixing effect in the strong confinement regime^33^, a distinct mechanism must be at play in CsPbBr_3_ QDs. Specifically, an Arrhenius fit to the corresponding temperature-dependent decay rate returns an activation energy of *E*_a_ = 9 ± 1 meV (Fig. 3d, inset), which is well within the broad phonon density of states in perovskite QDs^41^, suggesting an important role of phonons for the lifetime lengthening. At cryogenic temperatures, the entire oscillator strength is accumulated in the lowest-energy bright exciton state (superradiant state). By increasing the temperature, exciton–phonon coupling and the resulting disorder increase, such that the exciton population is transferred from the superradiant state to dark or less bright excited exciton states. As the radiative decay time is directly related to the oscillator strength of the state whose polarization has a constant phase across the QD, corresponding to the *k* = 0 transition (*k*, quasi-momentum), disorder- and phonon-induced exciton scattering processes thus quench the excitonic superradiant decay^30^. This peculiar temperature behaviour is a distinctive feature of superradiant systems and has been reported in, for instance, CuCl microcrystallites^25,26^, CdS microcrystallites^27^ and molecular J-aggregates^30^. This is further confirmed by the thermally induced wavefunction localization and dephasing obtained by ab initio molecular dynamics (AIMD) calculations (Extended Data Figs. 3b–g and  4, Supplementary Information section III and Supplementary Videos 1 and 2).

## Composition-dependent exciton lifetime

**Fig. 4:**
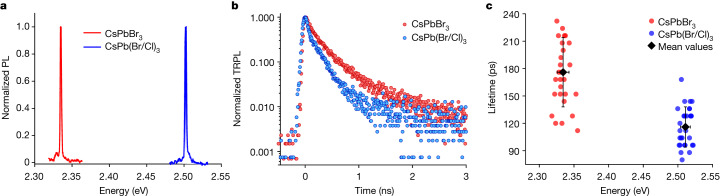
Composition-dependent exciton lifetime. **a**, Representative photoluminescence spectrum of a 30-nm CsPbBr_3_ QD (red line) and a 30-nm CsPb(Br/Cl)_3_ QD (blue line). **b**, TRPL decay for the QDs shown in **a**. **c**, Statistics over many different single QDs showing a net decrease in the radiative lifetime in CsPb(Br/Cl)_3_ QDs (blue points) compared with CsPbBr_3_ QDs (red points). For CsPb(Br/Cl)_3_ QDs, a minimum lifetime of 80 ps was achieved.

Apart from QD size and temperature, there are other parameters relevant for the acceleration of the radiative decay by excitonic SPS, for example, the Bohr diameter. As pointed out in ref. ^3^, a stronger enhancement of the oscillator strength in relation to the extent of wavefunction delocalization could be obtained in a material with a smaller Bohr radius, such as CsPb(Br/Cl)_3_, enabling relatively larger exciton delocalization for a given QD size^5^. Consequently, we explore weakly confined 30 nm CsPbBr_3_ QDs and CsPb(Br/Cl)_3_ QDs, the latter being obtained by halide exchange from their 30 nm CsPbBr_3_ counterpart (for details, see Supplementary Information). Halide exchange induces a blue shift of the exciton photoluminescence from 2.335 eV for the pure bromide to about 2.502 eV for the mixed chloride–bromide QD (Fig. 4a).

In Fig. 4b, representative decay traces of CsPbBr_3_ QDs and CsPb(Br/Cl)_3_ QDs establish the increased decay rate obtained through halide exchange. Whereas the mean lifetime is 180 ± 40 ps for CsPbBr_3_ QDs, similarly sized CsPb(Br/Cl)_3_ QDs feature a mean lifetime of 120 ± 20 ps (Fig. 4c). Compared with the 23 nm CsPbBr_3_ QDs discussed in Fig. 2, the lifetime is only weakly accelerated in these slightly larger 30-nm CsPbBr_3_ QDs. This suggests that for CsPbBr_3_, the QDs already reached a size regime for which the coherence volume is no longer limited by the QD size but more probably by the exciton–phonon coupling at this finite temperature (4 K). By contrast, a further increased wavefunction delocalization is obtained by composition tuning from CsPbBr_3_ to CsPb(Br/Cl)_3_ with 30-nm QDs, as inferred from the notably shortened lifetimes in the mixed-halide QDs. The observed enhancement of the decay rate after the reduction of the Bohr radius through halide exchange further corroborates the observation of excitonic SPS. Around 30% of QDs feature a sub-100 ps lifetime with the fastest lifetime down to 80 ps, making perovskite QDs one of the fastest cavity-free single-photon solid-state emitters studied so far.

## Discussion

**Extended Data Fig. 5:**
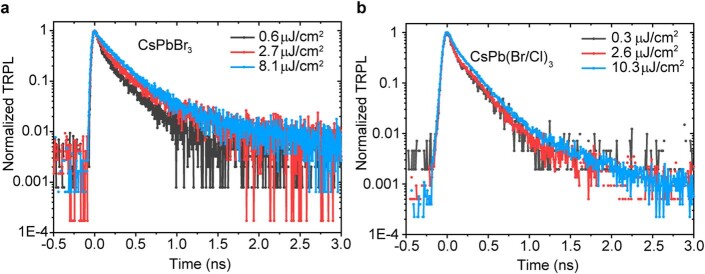
Fluence-dependent exciton lifetime. TRPL decay of a 30 nm CsPbBr_3_ (**a**) and CsPb(Br/Cl)_3_ (**b**) QD at different excitation fluences, respectively. The exciton lifetime is essentially fluence-independent. The discernable small lifetime elongation under strong laser illumination is attributed to a local heating effect induced by the laser spot.

**Extended Data Fig. 6:**
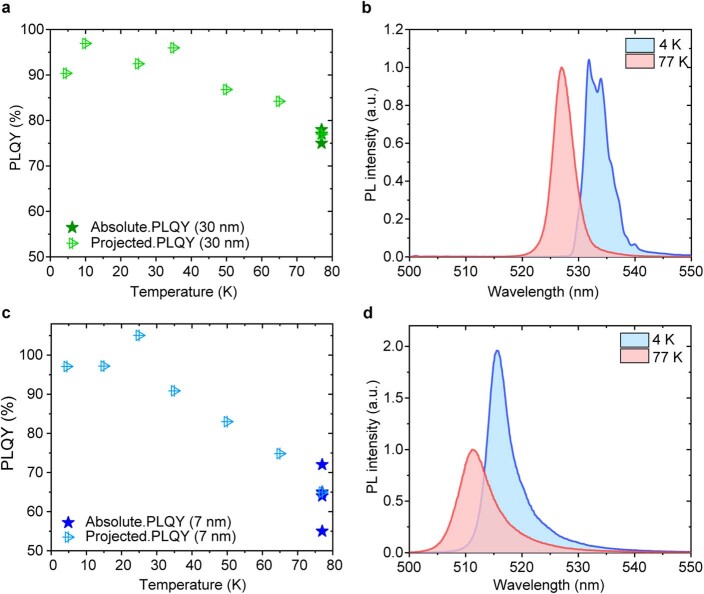
Temperature-dependent PLQY of large (30 nm) and small (7 nm) CsPbBr_3_ QDs. **a**,**c**, Evolution of the PLQY of an ensemble of 30 nm and 7 nm CsPbBr_3_ QDs, respectively. The absolute PLQY was measured at 77 K and projected to lower temperatures via the relative PL intensity ratio. Both samples show over 90% PLQY at 4 K. **b**,**d**, The PL spectra at 4 K and 77 K for the two studied samples.

Size-, temperature-, and composition-dependent single-QD photoluminescence lifetime measurements comprise compelling experimental evidence for the proposed excitonic SPS mechanism. To assign the observed decay rate enhancement to excitonic SPS, we need to additionally (1) confirm that the speed-up can be attributed to an enhancement of the radiative decay rate and (2) address the single-quantum nature of the emitting species, that is, single-photon emission. To verify that the decay rate can be attributed to radiative decay, we first studied the fluence-dependent photoluminescence lifetime of single CsPbX_3_ QDs. As shown in Extended Data Fig. 5, the photoluminescence lifetime is fluence-independent over two orders of magnitude (0.3–10.3 μJ cm^−2^), suggesting that the short lifetime is not dominated by fluence-dependent non-radiative recombination mechanisms, for example, Auger non-radiative processes^42,43^. Second, to exclude influences from other possible non-radiative recombination, for example, charge-carrier trapping, we probe the photoluminescence quantum yield (PLQY) at various temperatures. As shown in Extended Data Fig. 6, the projected PLQY at 4 K reaches more than 90%, in line with previously reported data^4,11^, confirming that the speed-up of the exciton decay is dominated by accelerated radiative recombination. This near-unity PLQY reaffirms that the radiative decay is from the bright state with negligible influence from dark excitons.

**Extended Data Fig. 7:**
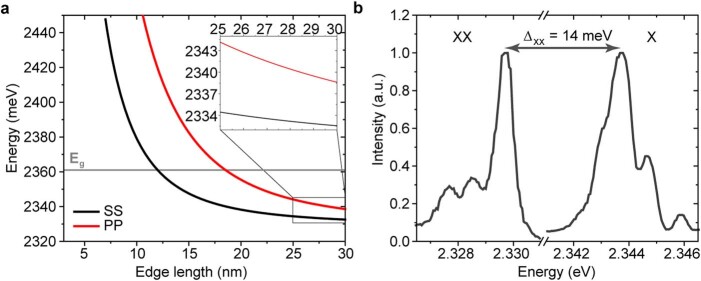
Excitonic optical transitions in CsPbBr_3_ QDs. **a**, Predicted size dependent energy for the S-to-S (SS, black line) and P-to-P (PP, red line) transitions. For the large 30 nm QD, the energy difference between SS and PP amounts to ca. 5 meV, *i.e*., much larger than the thermal energy at 4 K. **b**, PL spectrum for large 30 nm QDs showing a sizeable biexciton binding energy on the order of ca. 14 meV.

Next, we demonstrate that the large QDs are quantum emitters with discrete energy levels. In solid-state quantum emitters, the blockade mechanism responsible for the generation of on-demand single photons is associated with the discretization of the energy levels and the Pauli exclusion mechanism, resulting in an effective two-level system with a saturable absorption. These large QDs are becoming more bulk-like and therefore could arguably behave as classical rather than quantum light sources, thus contradicting the suggested SPS mechanism. Therefore, we calculated the size-dependent energy for the S-to-S and P-to-P transitions (Extended Data Fig. 7). For the largest 30-nm QDs we explored, the energy difference between the S-to-S and P-to-P transitions still amounts to about 5 meV, that is, much larger than the thermal energy at 4 K, suggesting that we are still studying quantum emitters with discrete energy levels rather than a bulk material with a continuous density of states.

**Fig. 5:**
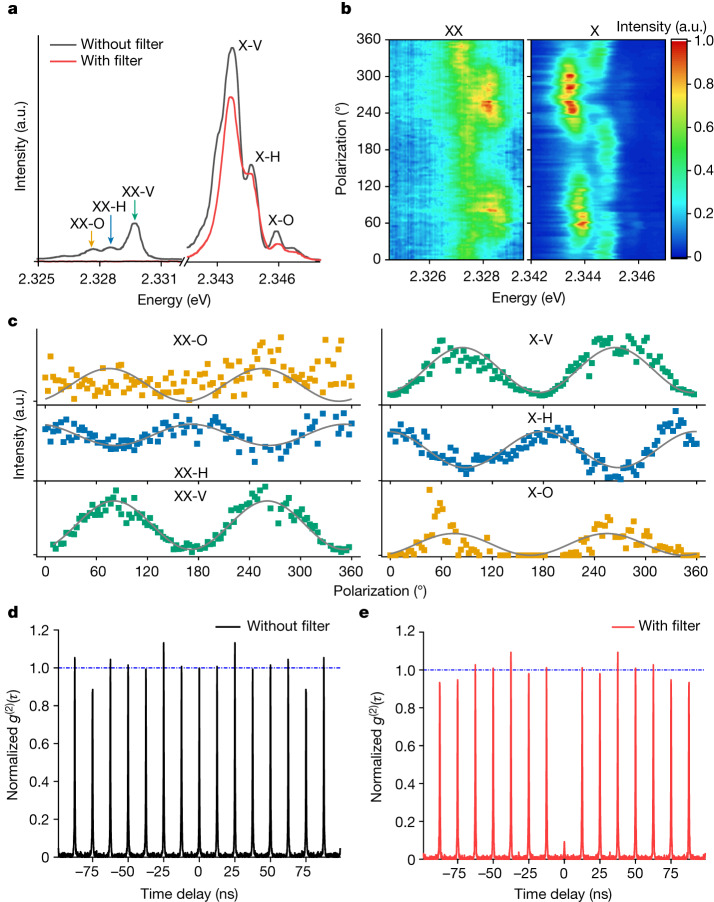
Single-photon emission from a 30-nm CsPbBr_3_ QD. **a**, Single-QD photoluminescence spectrum with (red) and without (black) a tunable long-pass filter rejecting biexciton emission. In the unfiltered spectrum, for each the exciton (X) and biexciton (XX), three sublevels are well resolved, denoted as V, H and O, respectively, with an average fine-structure splitting of 1.06 meV. **b**, Polarization-resolved photoluminescence spectra of biexciton and exciton, respectively. **c**, Intensity of the biexciton (left) and exciton sublevels (right) as a function of polarization angle, extracted by performing three-Lorentzian fitting on the spectra in **b**. The grey lines show a sin^2^ fit to the respective linearly polarized dipoles in the observation plane. A low signal-to-noise ratio for the XX-O state precludes a precise assignment of its polarization characteristics. **d**, Second-order intensity correlation *g*^(2)^(*τ*) acquired without spectral filtering. **e**, Second-order intensity correlation *g*^(2)^(*τ*) acquired after filtering out biexciton emission. A strong anti-bunching *g*^(2)^(0) < 0.5 is observed, attesting to single-photon emission from exciton recombination.

Then, we explain the optical properties of band-edge excitons, spectrally identifying excitons and biexcitons in weakly confined QDs. At high excitation fluence (0.85 μJ cm^−2^), the photoluminescence spectrum acquired with high spectral resolution (0.3 meV) features several emission peak manifolds that are assigned to the fine structure of exciton and biexciton, at high and low emission energies, respectively (Fig. 5a, dark solid line). To confirm this attribution, we measured the polarization of exciton and biexciton fine structures. As shown in Fig. 5a, exciton (X) and biexciton (XX) triplets were observed with a fine structure splitting (*∆*_FFS_ = ((*E*_2_ − *E*_1_) + (*E*_3_ − *E*_2_))/2) of around 1.06 meV for both species. The triplet sublevels are denoted as H, V and O, respectively, representing their individual polarization orientation. Figure 5b shows the spectrally and polarization-resolved detection of each sublevel for both the biexciton (transitions |XX⟩ to |X⟩) and exciton (transitions |X⟩ to |G⟩), showing a mirror-symmetric correlation in intensities of the sublevels with respect to the linear polarizer angles. This can further be shown by the integrated intensity of each sublevel of the exciton and biexciton triplet, plotted as a function of the polarizer angle in Fig. 5c. Each sublevel possesses a linear polarization profile, attested by the agreement with a sin^2^ function (fitted grey line), with crossed polarization orientations, in agreement with previous findings^11,13,44–46^. Moreover, the total energy and polarization preserves for each set of biexciton–exciton peaks as a result of the two-photon cascade decay from the biexciton state (with zero angular momentum) through the exciton state to the ground state^46–48^. All these experiments show that radiative speed-up can be achieved in a quantum system with discrete energy levels in all dimensions.

**Extended Data Fig. 8:**
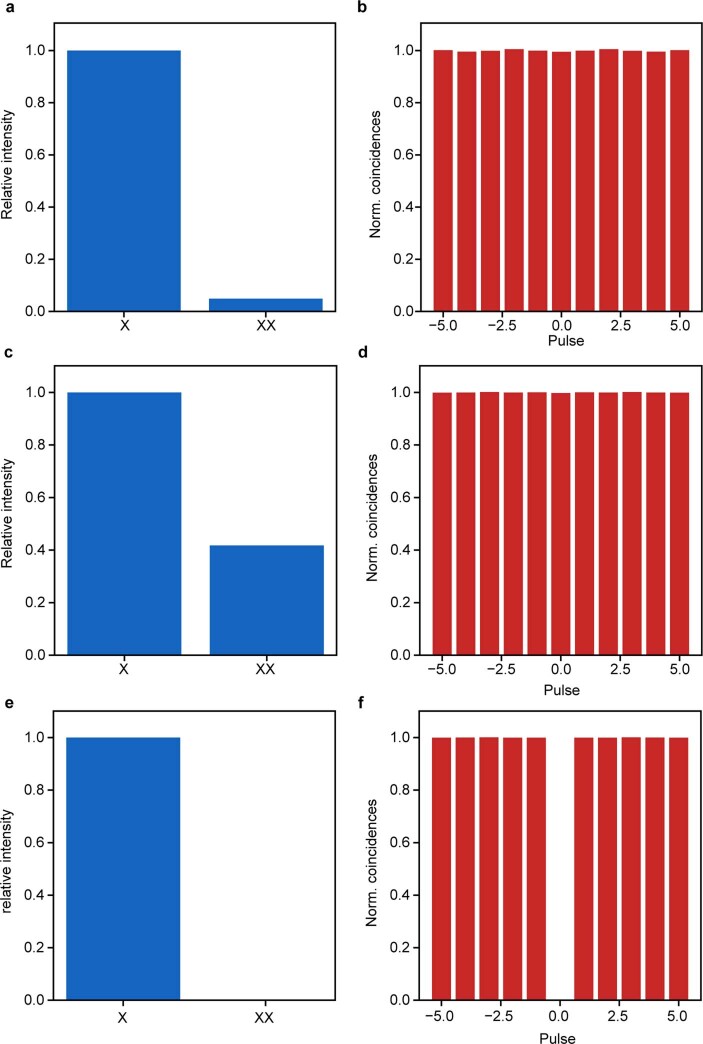
Monte Carlo simulations of the photon statistics of exciton (X) and biexciton (XX) emission. **a**, Relative intensity of X and XX for a mean number of excitons per pulse of <N>=0.1 and 100% photoluminescence quantum yield (PLQY) for both X and multiexcitons. **b**, Simulated g^(2)^ of a photon stream following the photon statistics from **a** attesting the absence of anti-bunching behaviour. **c,d** Analogous simulation of photon statistics and g^(2)^ for <N>=1 and 100% PLQY for both X and multiexcitons approximately recovering the intensity ratio and weak antibunching observed in our experiments. **e,f** Analogous simulation of photon statistics and g^(2)^ for <N>=1 and 0% PLQY for multiexcitons, attesting that simulations recover the anti-bunching that was observed after filtering out the biexciton emission.

Next, we show the quantum nature of the emitted photon stream by recording the photon statistics using second-order correlation (*g*^(2)^(*τ*)) measurements in a Hanbury-Brown and Twiss (HBT) set-up (Fig. 5d,e). Without spectral filtering, no anti-bunched emission was observed, that is*, g*^(2)^(*τ* = 0) ≈ 1, suggesting near-Poissonian photon statistics. Because *g*^(2)^(*τ* = 0) scales with the ratio of biexciton to exciton quantum yields^49^, this indicates the presence of efficient radiative biexciton recombination, a characteristic of these large QDs^12,49,50^ readily inferred from the strong biexciton peaks (Fig. 5a, black line). The experimental findings are corroborated by Monte Carlo simulations (see Extended Data Fig. 8 and discussion in Supplementary Information section IV). Introducing a tunable long-pass filter in the detection path suppresses the biexciton peaks (Fig. 5a, red line). Figure 5e shows the corresponding *g*^(2)^(*τ*), exhibiting a strongly anti-bunched peak at zero-time delay and hence confirming single-photon emission from the exciton recombination of weekly confined QDs. The observation of anti-bunching in the long-pass filtered emission further confirms our assignment of these red-shifted peaks (14 meV below the exciton; see Extended Data Fig. 7b) to the biexciton emission. By addressing the biexciton binding energies and the single-photon emission from exciton recombination in the weakly confined QDs, we confirm that these large QDs still behave as a quantum emitter, for which we established that their radiative lifetime exhibits dependencies characteristic of excitonic SPS.

## Conclusion

Exploiting the fine control over exciton QD sizes in highly engineerable colloidal perovskite QDs, we report the observation of excitonic SPS in a scalable and integrable material platform. Experimentally, we provide a comprehensive and robust picture of this cooperative process based on size-, composition-, and temperature-dependent radiative rates, corroborated by advanced *k* · *p*/effective mass theory calculations and AIMD simulations. Photoluminescence from weakly confined single CsPbX_3_ QDs shows a size-dependent acceleration of the exciton radiative decay, with radiative lifetimes approaching the reported exciton coherence times. These newly developed bright and coherent quantum light sources are bare nano probes (embedded neither in optical microcavities nor in dielectric matrices) that could be further engineered and used in quantum imaging applications. Furthermore, the rich features observed in those previously unexplored weakly confined excitons, such as strong electron–hole correlations that are at the heart of the current discovery, are going to be transformative in diverse fields of applications.

## Methods

### Synthesis

The 7–13 nm QDs were synthesized following the method reported in ref. ^51^. The 16–30 nm QDs were synthesized following the method reported in ref. ^52^. More details and samples characterization are provided in Supplementary Information.

### Sample preparation

#### For single-QD spectroscopy measurement

The preparation consisted of two main steps: dilution and spin coating. For QDs with the size of 7–16 nm, dispersed in toluene, the dispersion of QDs with a concentration of about 1 mg ml^−1^ was diluted by a factor of 100 in toluene (ACROS, 99.85% extra dry over molecular sieves). The solution was further diluted by another factor of 100 in a 3-mass% solution of polystyrene (ALDRICH, average *M*_w_ ≈ 280,000) in toluene. The final solution (50 μl) was spin-coated onto an intrinsic crystalline Si wafer with a 3-μm-thick thermal-oxide layer at 50 revolutions per second for 60 s.

For QDs with the size of 23–30 nm, dispersed in the mixture (1:1) of toluene (ACROS, 99.85% extra dry over molecular sieves) and 1,2-dichlorobenzene (ALDRICH, anhydrous, 99%), the dispersion of QDs with a concentration of about 1 mg ml^−1^ was diluted by a factor 80 in mixture (1:1) of toluene and 1,2-dichlorobenzene. The solution was further diluted by another factor of 100 in a 3-mass% solution of polystyrene (ALDRICH, average *M*_w_ ≈ 280,000) in toluene. The final solution (50  μl) was spin-coated onto an intrinsic crystalline Si wafer with a 3-μm-thick thermal-oxide layer at 50 revolutions per second for 60 s.

#### For PLQY measurements

The colloidal dispersion (0.1  ml) was drop-casted on a glass substrate for absolute PLQY measurement through Quantaurus QY (C11347-11, Hamamatsu) at room temperature and liquid nitrogen temperature (77 K).

The colloidal dispersion (0.1 ml) was drop-casted on a crystalline Si wafer with a 3-μm-thick thermal-oxide layer for relative PLQY measurement through our μ-PL set-up.

All the above-mentioned sample preparations were finished in a glove box under an N_2_ atmosphere.

##### Optical characterization

For single-QD spectroscopy, a custom-built μ-PL set-up was used. The samples were mounted on *xyz* nano-positioning stages inside an evacuated liquid-helium closed-loop cryostat (Montana Instruments) and cooled down to a targeted temperature of 4 K. Single QDs were excited using a fibre-coupled excitation laser, which is focused (1/e^2^ diameter = 2.4 μm) on the sample by a microscope objective (NA = 0.8, 100×). Typical power densities used to excite single QDs were 0.36–5.7 μJ cm^−2^. The emitted light was collected by the same objective and passed through a dichroic mirror (long-pass, cut-on wavelength 450 nm long-pass filter at 450 nm). A monochromator coupled to an EMCCD (Princeton Instruments, 0.5-m, 1 s binning time) was used for spectra measurements. A single APD (MPD, time resolution of 50 ps) mounted after the monochromator, which accepts photons only from the exciton photoluminescence, was used to measure TRPL traces. A HBT set-up with a 50/50 beam splitter, two APDs and a TCSPC Module (PicoQuant) was used for second-order correlation (*g*^(2)^(*τ*)) measurements. For *g*^(2)^(*τ*) measurements, QDs were excited with an excitation fluence of around 0.85 μJ cm^−^^2^. To filter out the biexciton emission, we used a tunable short pass filter (Semrock, F35-559, cut-on wavelength was tunable between 496 nm and 565 nm). Photoluminescence spectra can be measured with a grating of 300 lines per mm, blaze at 500 nm, giving around 1 meV spectral resolution or with high spectral resolution (approximately 0.3 meV) by using a grating with 1,800 lines per mm and blaze at 500 nm. Polarization-resolved spectra were measured under 1.5 μJ cm^−2^ excitation, with a grating of 1,800 lines per mm, blaze at 500 nm and a linear polarizer in the collection path with the rotatable axis.

##### AIMD simulations

Density functional theory simulations were performed based on previously reported geometry optimized CsPbBr_3_ and CsPbBr_3_/CsCaBr_3_ QD models^41^ in vacuum (at least 1 nm on each side). Electronic structure calculations and molecular dynamics simulations were performed in CP2K (ref. ^53^) using the Quickstep module with Gaussian and plane waves featuring a plane-wave cutoff of 300 Ry (ref. ^54^). We used DZVP-MOLOPT basis sets, Goedecker–Teter–Hutter pseudopotentials^55–57^ and Perdew–Burker–Enzerhof exchange-correlation functionals^58^.

Molecular dynamics simulations were performed within the NVT ensemble using a canonical sampling through velocity rescaling thermostat^59^ with a time constant of 15 fs at the respective temperatures or in the NVE ensemble after thermal equilibration. Molecular dynamics trajectories of length 20  ps were simulated with 1 fs or 10 fs timesteps and at least the first 6 ps were considered as equilibration. After equilibration, we quantified the localization of the highest occupied molecular orbital (HOMO) wavefunction every 0.1 ps using the cube files. The inverse participation ratio (IPR) of the associated probability density $\rho (r)={|\Psi (r)|}^{2}$ was obtained from

1 $${\rm{IPR}}=\frac{\int \rho {(r)}^{2}{\rm{d}}r}{{(\int \rho (r){\rm{d}}r)}^{2}}$$

and then time-averaged along the trajectory. Simultaneously, root-mean-squared displacement of the centre of mass of the HOMO density from the QD centre of mass was obtained and then time-averaged.

Time-dependent dephasing functions *D*(*t*, *T*) were obtained from the optical response formalism^60^

2 $$D(t,T)={{\rm{e}}}^{-g(t,T)}$$

where *g*(*t*, *T*) is the lineshape function

3 $$g(t,T)=\frac{1}{{\hbar }^{2}}{\int }_{0}^{t}{\rm{d}}{\tau }_{1}{\int }_{0}^{{\tau }_{1}}{\rm{d}}{\tau }_{2}C({\tau }_{2},T)$$

which is obtained from $$C({\tau }_{2},T)=\langle \Delta {E}_{{\rm{HOMO}}}({\tau }_{2},T)\Delta {E}_{{\rm{HOMO}}}(0,T)\rangle $$, the autocorrelation function of the HOMO energy fluctuation $$\Delta {E}_{{\rm{HOMO}}}({\tau }_{2},T)\,=$$$${E}_{{\rm{HOMO}}}({\tau }_{2},T)-\langle {E}_{{\rm{HOMO}}}({\tau }_{2},T)\rangle $$, where ⟨…⟩ denotes time-averaging. More details are provided in Supplementary Information section III.

##### Simulation of photon statistics

Monte Carlo simulations of (multi-)exciton emission and of the HBT experiments were performed in Python using the pycorrelate package (https://github.com/tritemio/pycorrelate). Excitations were randomly created in the QD with a Poissonian exciton number distribution characterized by its mean number of excitations ⟨*N*⟩. Exciton and multiexciton quantum yields were set to 1 because of the high PLQY of the samples and weak confinement of large QDs. The generated stream of 0–4 photons (higher excitations negligible) was then analysed in an HBT experiment using a Monte Carlo approach yielding the arrival times on the two detectors. To obtain the second-order correlation function *g*^(2)^(*τ*), we applied the pcorrelate function of the pycorrelate package to the simulated photon arrival times. Note that the exclusion of noise and losses would further weaken the anti-bunching because of their Poissonian nature. More details are given in Supplementary Information section IV.

## Online content

Any methods, additional references, Nature Portfolio reporting summaries, source data, extended data, supplementary information, acknowledgements, peer review information; details of author contributions and competing interests; and statements of data and code availability are available at 10.1038/s41586-023-07001-8.

### Supplementary information

Supplementary Information This file contains Supplementary Figs. 1–5 and Tables 1–3.

Supplementary Video 1 AIMD calculations of the time-resolved (0.1 ps resolution) HOMO (highest occupied molecular orbital) density variation for core-only QDs at 3 K and 150 K, respectively, showing thermally induced localization of the HOMO density.

Supplementary Video 2 AIMD calculations of the time-resolved (0.1 ps resolution) LUMO (least unoccupied molecular orbital) density variation for core-shell QDs at 3 K and 150 K, respectively, showing thermally induced localization of the LUMO density.

## Data Availability

The datasets generated during and/or analysed during this study are available in a repository at the ETH Library (10.3929/ethz-b-000646273).
