# Supplementary material for: Single-photon superradiance in individual caesium lead halide quantum dots
Source: Nature. 2024 Jan 31;626(7999):535–41. doi: 10.1038/s41586-023-07001-8 (PMC10866711; doi:10.1038/s41586-023-07001-8)
Supplement: Supplementary file 1 — This file contains Supplementary Figs. 1–5 and Tables 1–3. [file 41586_2023_7001_MOESM1_ESM.pdf]

nature portfolio

https://doi.org/10.1038/s41586-023-07001-8

Supplementary information

Single-photon superradiance in individual caesium lead halide quantum dots

In the format provided by the
authors and unedited

Supplementary Information

Single-photon superradiance in individual cesium lead halide quantum dots

Chenglian Zhu,[1,2] *Simon C. Boehme,*[1,2] *Leon G. Feld,*[1,2] *Anastasiia Moskalenko,*[1,2] *Dmitry N. Dirin,*[1,2] *Rainer F. Mahrt,*[3] *Thilo Stöferle,*[3] *Maryna I. Bodnarchuk,*[2] *Alexander L. Efros,*[4] *Peter C. Sercel,*[5*,] *Maksym V. Kovalenko*[1,2,*] *and Gabriele Rainò*[1,2,*]

[1] Institute of Inorganic Chemistry, Department of Chemistry and Applied Biosciences, ETH Zürich, CH-8093 Zürich, Switzerland

[2] Laboratory for Thin Films and Photovoltaics, Empa – Swiss Federal Laboratories for Materials Science and Technology, CH-8600 Dübendorf, Switzerland

[3] IBM Research Europe – Zurich, Säumerstrasse 4, 8803 Rüschlikon, Switzerland.

[4] Center for Computational Materials Science, Naval Research Laboratory, Washington DC 20375 USA

[5] Center for Hybrid Organic Inorganic Semiconductors for Energy, Golden, CO 80401, USA.

CONTENTS

I. Sample synthesis 3
 A. Synthesis of 7-13 nm $CsPbBr_3$ QDs 3
 B. Synthesis of 16-30 nm $CsPbBr_3$ QDs 4
 C. Anion exchange on the 30 nm $CsPbBr_3$ QDs 6

II. Theoretical calculations 6
 A. Quantum confinement model for excitons in QDs 6
 B. Low temperature photoluminescence decay lifetime 15
 C. Connection to Dicke's theory of superradiance 24

III. *Ab-initio* molecular dynamics simulations 33

IV. Photon statistics of exciton and biexciton emission 40

References 43

I. Sample synthesis

A. Synthesis of 7-13 nm $CsPbBr_3$ QDs

The synthesis of 7 nm, 9 nm, and 13 nm $CsPbBr_3$ QDs was performed according to the method reported in Ref.1 and is described in detail here below.

Chemicals.

Lead(II) acetate trihydrate, oleic acid (OA, 90%), cesium carbonate, bromine, 1-octadecene (ODE), 3-(N,N-dimethyloctadecylammonio) propanesulfonate (ASC18, >99% for 9 nm and 13 nm), soy-lecithin (>97%, biochemistry grade) from Roth (for 7 nm), and toluene were purchased from Sigma Aldrich, trioctylphosphine (TOP) from STREM, ethyl acetate and acetone from Fischer.

Synthesis.

Cs-oleate 0.4 M precursor solution in ODE is prepared by dissolving Cs_2CO_3 (1.628 g, 5 mmol) in oleic acid (OA, 5 mL, 16 mmol) and ODE (20 mL) upon evacuating it at 120 °C until the completion of gas evolution. Analogously, Pb-oleate 0.5 M stock solution in ODE is prepared by dissolving $Pb(CH_3COO)*3H_2O$ (4.607 g, 12 mmol) in OA (7.6 mL, 24 mmol) and ODE (16.4 mL). TOP-Br_2 0.5 M stock solution is prepared by mixing TOP (7 mL, 15 mmol) with toluene (18.7 mL) and bromine (0.6 mL, 11.5 mmol) under inert atmosphere and vigorous stirring.

For a synthesis of 7 nm large $CsPbBr_3$ QDs, lecithin (1.305 mg) was mixed with 10 ml ODE, 16 ml Cs-oleate, and 20 ml Pb-oleate stock solutions. The reaction vessel was purged 3 times and heated under inert gas to 80 °C, followed by the injection of 20 ml $TOPBr_2$ stock solution. The reaction was immediate, and the resulting crude solution was cooled to room

temperature using a water-ice bath. The crude solution was centrifuged at 20133 g at 17 °C for 10 minutes.

A part of the obtained supernatant (20 ml) was used for farther size-selection of QDs. Anhydrous acetone (9 ml) was added to the solution of QDs, followed by centrifugation at 20133 g at 17 °C for 10 minutes. QDs from the obtained supernatant were progressively precipitated by extra 1 ml of anhydrous acetone and the obtained pellets were redissolved in 1 ml of anhydrous toluene each, resulting in a series of fractions of QDs with decreasing sizes. Fraction #4 (corresponding to 12 ml acetone total) has been used in this work without additional purification.

For a synthesis of 9 and 13 nm $CsPbBr_3$ QDs, ASC18 (100 mg), was mixed with 23 ml ODE, 1.8 ml Cs-oleate and 2.3 ml Pb-oleate stock solutions. The reaction vessel was purged 3 times and heated under inert gas to 180 °C, followed by the injection of 2.3 ml $TOPBr_2$ stock solution. The reaction was immediate, and the resulting crude solution was cooled to room temperature using a water-ice bath. The crude solution was centrifuged at 29464 g for 10 minutes. The precipitate was redispersed in 1 mL of toluene and centrifuged for 10 more minutes. The latest was repeated once again resulting in a solution of 13 nm large QDs. Smaller (9 nm) QDs have been precipitated from the supernatant of the crude solution by adding 46 ml of ethyl acetate, centrifugation at 29464 g for 2 minutes and subsequent redispersion in 1 mL of toluene. The final QD dispersion was centrifuged at 9740 g for 2 minutes again in order to remove larger QDs.

B. Synthesis of 16-30 nm $CsPbBr_3$ QDs

The synthesis of such $CsPbBr_3$ QDs was conducted according to the Ref.2 with small modifications, as described below.

Preparation of the Cs oleate precursor, 0.06 M.

In a typical synthesis, 1.2 mmol (195,4 mg) of Cs_2CO_3 was loaded in a 25 mL three-neck round-bottom flask along with 9 mL of 1-octadecene (ODE) and 1 mL of oleic acid (OA). The reaction mixture was degassed with nitrogen for 1 h at 120 °C, then the temperature was increased to 150 °C forming a clear Cs-oleate solution, and annealed at that temperature for another 10 min. The synthesis is accompanied by purging nitrogen into the reaction mixture all the time. The solution was stored in a glovebox.

In a typical synthesis, 0.2 mmol of PbO (44.6 mg), 0.6 mmol of phenacyl bromide (119.4 mg), 1 mL of OA, and 5 mL of ODE were loaded in a 25 mL round-bottomed flask, and deaerated at 120 °C for 1 h by purging nitrogen gas directly into the solution. Then the temperature was increased to 220 °C and 0.6 mL oleylamine was injected. The solution became red initially within 1 min, and then gradually turned to bright yellow around 15 min.

The reaction mixture was cooled down to 210 °C (in the case of 16 nm QDs, 220 °C – 23 nm and 225 °C - 30 nm) and 0.5 mL of Cs-oleate was injected swiftly into the bright yellow solution at 210 °C. The solution was annealed for 60 minutes at that temperature. Then, the reaction mixture was cooled down to room temperature with an ice bath.

The crude solution was centrifuged for 5 min at 6000 rpm. The resulting supernatant was discarded, and the precipitate was dispersed in toluene (2 mL). The solution was centrifuged a second time for 3 min at 6000 rpm and the precipitate was discarded.

In order to grow larger QDs, the injection of Cs-oleate and annealing were conducted at 220 °C (for 23 nm QDs) or 225 °C (for ~ 30 nm QDs).

QDs larger as 23 nm can be colloidally stabilized by lecithin. While the reaction mixture was cooling down, at 50 °C 2 ml of lecithin in toluene (0.01 M) were added and then solution was stirred for the next 30 min. Then the crude solution was centrifuged for 5 min at 3000 rpm. The resulting supernatant was discarded, and the precipitate was dispersed in a lecithin/toluene mixture (0,01 M, 2 mL) and kept undisturbed for 1 hour. Then, the solution was centrifuged a second time for 4 min at 3000 rpm. The precipitate was redispersed in toluene/1,2-dichlorobenzene (v/v 0.6:1).

C. Anion exchange on the 30 nm $CsPbBr_3$ QDs

30 nm $CsPbBr_3$ QDs (0.2 mL, prepared as described above) were converted into $CsPb(Br/Cl)_3$ QDs by the treatment with didodecyldimethylammonium chloride (DDAC)/$PbCl_2$ in toluene (6.5 uL). The solution of DDAC/$PbCl_2$ in toluene was prepared by mixing 42 mg DDAC, 13.8 mg PbCl2 with 7.5 mL toluene under moderate heating.

II. Theoretical calculations

A. Quantum confinement model for excitons in QDs

Here we review previously derived expressions for the size dependence of the lowest energy exciton in cuboidal/spheroidal lead halide perovskite (LHP) quantum dots (QDs). Since the conduction and valence bands in bulk LHP are each two-fold degenerate simple bands[3], a description of the confined excitons is obtained in terms by solving the effective mass equation within the NC in a simple band description:

$$\hat{H}^{eff} f(\boldsymbol{r}_e, \boldsymbol{r}_h) = E_x\, f(\boldsymbol{r}_e, \boldsymbol{r}_h)\,, \qquad \text{(S1)}$$

where $f(\boldsymbol{r}_e, \boldsymbol{r}_h)$ is the envelope function for the exciton, written as a function of the electron and hole coordinates, r_e, r_h, respectively. E_x is the exciton energy, and the effective mass Hamiltonian for the electron/hole pair, $\hat{H}^{eff}$, is given by the sum of the band gap, E_g, the electron and hole kinetic energies, and their potential energy of interaction:

$$\hat{H}^{eff} = E_g - \frac{\hbar^2}{2m_e}\nabla_e^2 - \frac{\hbar^2}{2m_h}\nabla_h^2 - \frac{e^2}{\epsilon_{eff}|\boldsymbol{r}_e - \boldsymbol{r}_h|} \, . \tag{S2}$$

Here, ϵ_{eff} is an effective dielectric constant screening the electron/hole Coulomb interaction [4], while m_e and m_h are the electron and hole effective masses. This equation must be solved subject to the boundary condition that the envelope function vanishes on the QD surface. Since the exciton radius in LHPs such as $CsPbBr_3$ is $a_x \sim 3$ nm, smaller than or comparable to the QD size, we adopt an intermediate confinement description of the exciton[3,5-8], entailing a variational determination of the electron/hole Coulomb corrections to the single particle quantum confined energy levels. Writing the envelope function of the ground electron and hole quantum confined levels in cuboidal QD edge lengths L_x, L_y, L_z,

$$\psi_S(x,y,z) = \sqrt{\frac{8}{L_x L_y L_z}} \cos\left(\frac{\pi x}{L_x}\right) \cos\left(\frac{\pi y}{L_y}\right) \cos\left(\frac{\pi z}{L_z}\right) , \tag{S3}$$

the envelope function of the lowest energy exciton is assumed to be of the form,

$$f(\boldsymbol{r}_e, \boldsymbol{r}_h) = \frac{1}{\sqrt{\mathcal{N}(\beta)}} e^{-\beta|r_e - r_h|} \psi_S(\boldsymbol{r}_e) \psi_S(\boldsymbol{r}_h) \, . \tag{S4}$$

Here the terms involving the parameter β reflects the correlated motion of the electron and hole due to their Coulomb interaction. Using this ansatz function, the exciton energy E_x is calculated as,

$$E_x = \frac{\langle f|\hat{H}^{eff}|f\rangle}{\langle f|f\rangle}. \tag{S5}$$

The optimum variational parameter β_{opt} is determined as the value of β which minimizes the energy for a QD of given edge lengths, L_x, L_y, L_z.

Evaluating the energy, we find that it can be written in the form,

$$E_x = E_g - \frac{\hbar^2}{2\mu L_{eff}{}^2}\frac{I_K}{\mathcal{N}} + \frac{e^2}{\epsilon_{eff}\, L_{eff}}\ \frac{I_C}{\mathcal{N}} \tag{S6}$$

In this expression, $\mu = (1/m_e + 1/m_h)^{-1}$ is the reduced effective mass, while I_K, I_C are dimensionless integrals related to the kinetic and potential energies, and $\mathcal{N}$ is a normalization integral. These integrals are written in terms of dimensionless coordinates, $\boldsymbol{u}_e = \boldsymbol{r}_e/L_{eff}$ and $\boldsymbol{u}_h = \boldsymbol{r}_h/L_{eff,}$ where the effective length, $L_{eff,}$ is chosen as the edge length of a cube with the same kinetic energy for the lowest energy exciton given by Ref.7,

$$\left(\frac{3}{L_{eff}{}^2}\right) = \left(\frac{1}{L_x{}^2} + \frac{1}{L_y{}^2} + \frac{1}{L_z{}^2}\right) \tag{S7}$$

To perform the integrals, it is useful to express the wave function of the exciton in dimensionless form. Defining the ratios $l_x = L_x/L_{eff,}\, l_y = L_y/L_{eff}, l_z = L_z/L_{eff}$, we rewrite the ground exciton envelope function in terms of $\boldsymbol{u} = \boldsymbol{r}/L_{eff}$:

$$\psi_S(u_x, u_y u_z) = \sqrt{\frac{8}{l_x l_y l_z}} \cos\left(\frac{\pi u_x}{l_x}\right) \cos\left(\frac{\pi u_y}{l_y}\right) \cos\left(\frac{\pi u_z}{l_z}\right), \tag{S8}$$

$$f_{ss}(\boldsymbol{u}_e, \boldsymbol{u}_h) = \frac{1}{\sqrt{\mathcal{N}(b)}} e^{-b|\boldsymbol{u}_e - \boldsymbol{u}_h|} \psi_S(\boldsymbol{u}_e)\psi_S(\boldsymbol{u}_h)\,, \tag{S9}$$

where we have used $b = \beta\, L_{eff}$. Then the dimensionless kinetic energy integral becomes,

$$I_K = I_K(b) \qquad \text{(S10)}$$

$$= \int_{-\frac{l_x}{2}}^{\frac{l_x}{2}} \int_{-\frac{l_y}{2}}^{\frac{l_y}{2}} \int_{-\frac{l_z}{2}}^{\frac{l_z}{2}} d^3\,\boldsymbol{u}_e \int_{-\frac{l_x}{2}}^{\frac{l_x}{2}} \int_{-\frac{l_y}{2}}^{\frac{l_y}{2}} \int_{-\frac{l_z}{2}}^{\frac{l_z}{2}} d^3\,\boldsymbol{u}_h \;\; f_{ss}(\boldsymbol{u}_e, \boldsymbol{u}_h)\, \nabla_{\boldsymbol{u}_e}^2\, f_{ss}(\boldsymbol{u}_e, \boldsymbol{u}_h).$$

The Coulomb integral, I_C, is

$$I_C = I_C(b) \qquad \text{(S11)}$$

$$= \int_{-\frac{l_x}{2}}^{\frac{l_x}{2}} \int_{-\frac{l_y}{2}}^{\frac{l_y}{2}} \int_{-\frac{l_z}{2}}^{\frac{l_z}{2}} d^3\,\boldsymbol{u}_e \int_{-\frac{l_x}{2}}^{\frac{l_x}{2}} \int_{-\frac{l_y}{2}}^{\frac{l_y}{2}} \int_{-\frac{l_z}{2}}^{\frac{l_z}{2}} d^3\,\boldsymbol{u}_h \;\; \frac{1}{|\boldsymbol{u}_e - \boldsymbol{u}_h|}\, |f_{ss}(\boldsymbol{u}_e, \boldsymbol{u}_h)|^2,$$

while $\mathcal{N} = \mathcal{N}(b)$, the normalization integral, is written as,

$$\mathcal{N} = \mathcal{N}(b) \qquad \text{(S12)}$$

$$= \int_{-\frac{l_x}{2}}^{\frac{l_x}{2}} \int_{-\frac{l_y}{2}}^{\frac{l_y}{2}} \int_{-\frac{l_z}{2}}^{\frac{l_z}{2}} d^3\,\boldsymbol{u}_e \int_{-\frac{l_x}{2}}^{\frac{l_x}{2}} \int_{-\frac{l_y}{2}}^{\frac{l_y}{2}} \int_{-\frac{l_z}{2}}^{\frac{l_z}{2}} d^3\,\boldsymbol{u}_h \;\; \left|e^{-b|\boldsymbol{u}_e - \boldsymbol{u}_h|}\psi_S(\boldsymbol{u}_e)\psi_S(\boldsymbol{u}_h)\right|^2.$$

For reference, Fig. S1 shows the optimum value $b_{opt} = \beta_{opt}\, L_{eff}$ versus the ratio L_{eff}/a_x of the effective length to the bulk exciton radius. The plots shows that at large size, b_{opt} becomes linear in L; this is expected as we approach the weak confinement limit.

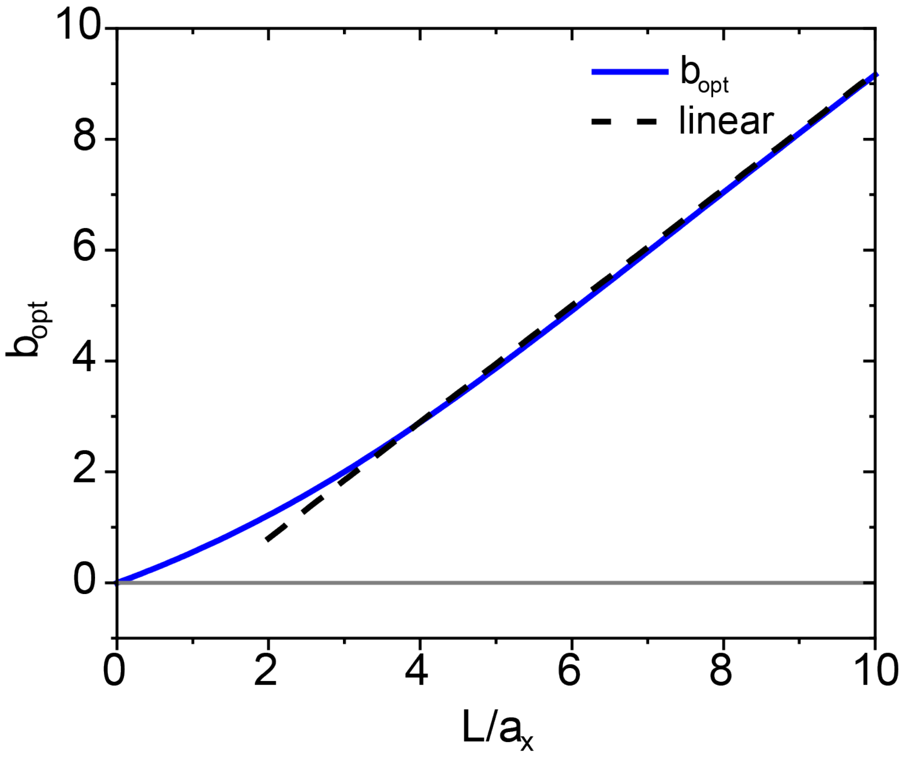

Fig. S1 | Size dependence of the optimum value of the variational parameter. Size dependence of the optimum value $b_{opt} = \beta_{opt}\, L_{eff}$ calculated for the exciton wavefunction in the intermediate confinement, Eq. S9, plotted versus the ratio L/a_x of the edge length, L, of a cube-shaped QD to the exciton radius, a_x. At large size, b_{opt} becomes linear in L/a_x.

The normalization integral $\mathcal{N}$ plays a key role in determining the exciton oscillator strength as we explore below. We will show for the large size limit, $b = \beta\, L_{eff} \gg 1$, that $\mathcal{N}(b)$ scales as $(a_{x/}L_{eff})^3$.

The simplest approach to applying Eq. S6 is within a parabolic band approximation, which is valid for QDs of size sufficiently large that the quantum confinement is not too large relative to the bandgap. Further simplifying to the case of a cube-shaped QD with edge length L, Eq. S6 can be recast in terms of the bulk exciton radius a_x and the bulk exciton binding energy B_x as,

$$E_x = E_g - \frac{B_x}{(L/a_x)^2}\left(I_K(b_{opt}(L)) + 2\frac{L}{a_x} I_C(b_{opt}(L))\right). \tag{S13}$$

This expression can be compared to the energy calculated within the strong and weak confinement limits[5], summarized in Table S1, which are valid respectively when the QD size is much less than or much greater than the bulk exciton radius as shown in Fig. S2. We see in Fig. S2 that the intermediate confinement approximation for the exciton energy smoothly transfers from the strong confinement result in the limit $L \ll a_x$ into the weak confinement result in the limit of large size ($L \gg a_x$). This suggests that the expression Eq. S4 for the exciton wavefunction derived for the intermediate regime can be used for the entire size range.

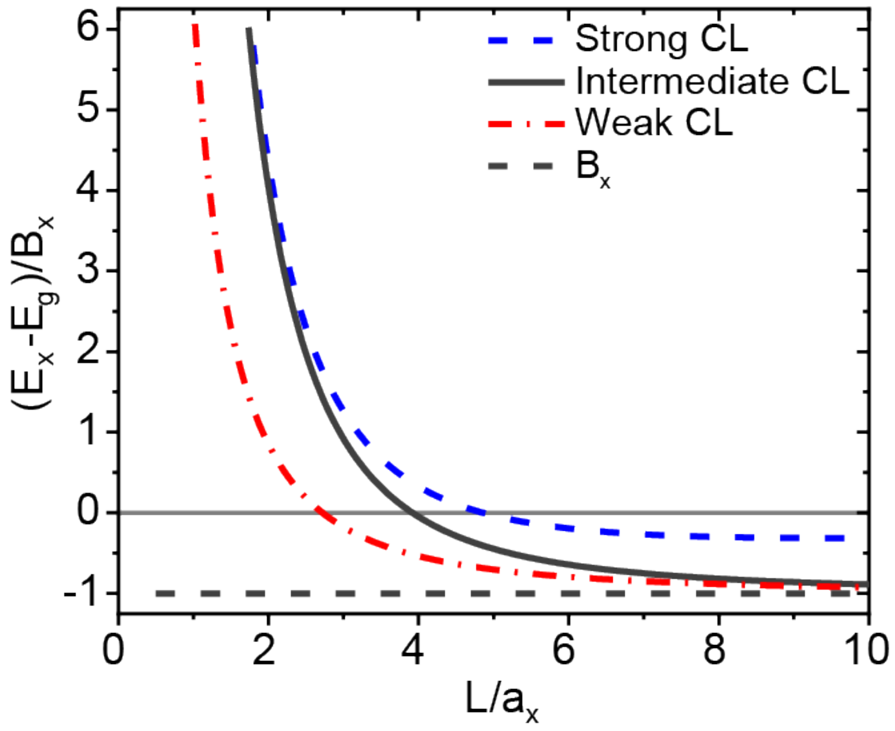

Fig. S2 | Exciton energy versus QD size. Exciton energy $E_x - E_g$ in units of the exciton binding energy, B_x, plotted versus the ratio L/a_x of the edge length, L, of a cube-shaped QD to the exciton radius, a_x. The solid black line represents the result of the variation calculation for the lowest energy S-to-S exciton state given by Eq. S13. For reference, the exciton energies calculated in the strong and weak confinement limit (blue dashed line, and red dash-dotted line, respectively) are shown as well as the bulk exciton binding energy (black dashed line).

Table S1. Exciton energy in the strong and weak confinement limits.

Energy of the lowest energy exciton for a cube-shaped QD of effective edge length L, in the strong and weak confinement limits (CL). The exciton radius, $a_x = a_o \epsilon_{eff}/\mu$ where a_o is the hydrogen Bohr radius, ϵ_{eff} is the effective dielectric constant, μ is the reduced effective mass, and the bulk exciton binding energy $B_x = \frac{\hbar^2}{2\mu a_x^2}$. The numerical factor in the expression for the strong confinement energy represents the numerical integration of the Coulomb energy within first-order perturbation theory.

	Lowest exciton energy
Strong confinement	$E_x = E_g + B_x \left\{ 3\left(\frac{\pi}{L/a_x}\right)^2 - 2\left(\frac{3.047}{L/a_x}\right) \right\}$
Weak confinement	$E_x = E_g + B_x \left\{ \frac{3}{4}\left(\frac{\pi}{L/a_x}\right)^2 - 1 \right\}$

In the calculations shown in Fig. 2b of the main text, Eq. S13 was evaluated using the exciton reduced effective mass and effective dielectric parameters determined by Yang *et al.*, Ref.9, for bulk thin films of $CsPbBr_3$ by analysis of exciton magneto-transmission measurements at 2 K. The bulk PL bandgap was treated as a fitting parameter. The resulting bulk exciton radius and bulk exciton binding energy were determined as 3.1 nm and 32 meV, respectively as summarized in Table S2.

This approach can be extended to describe higher energy exciton levels. For example, in addition to the ground exciton, formed from the S-like electron and S-like hole quantum size levels, in a cube-shaped NC there are 3 degenerate P-like to P-like exciton transitions. For these excitons, the carrier wavefunctions can be written in the form[4,7],

$$\psi_P(x,y,z) = \sqrt{\frac{8}{L^3}} \cos\left(\frac{\pi x}{L}\right) \cos\left(\frac{\pi y}{L}\right) \mathrm{Sin}\left(\frac{2\,\pi\, z}{L}\right), \tag{S14}$$

where the wavefunction has been written explicitly for the P_z -like state; and the exciton wavefunction is written using the ansatz[4,7],

$$f_{PP}(\boldsymbol{r}_e, \boldsymbol{r}_h) = \frac{1}{\sqrt{N(\beta_{PP})}} e^{-\beta_{PP}|\boldsymbol{r}_e - \boldsymbol{r}_h|} \psi_P(\boldsymbol{r}_e) \psi_P(\boldsymbol{r}_h) \,. \tag{S15}$$

The energy for these P-to-P states can be calculated using Eq. S6 by substituting ψ_P for ψ_S and f_{PP} for f_{SS} in Eqs S10-S12. A comparison of the exciton transition energies computed for the ground S-S and the excited P-P exciton transitions calculated in this manner is shown in **Extended Data Fig. 7 (a).**

It is instructive to compare Eq. S6 or equivalently Eq. S13, which include the effect of Coulomb-induced correlated motion of the electron and hole variationally, to the results obtained by many-body models of correlated single excitons. In Fig. S3a, we compare the size dependent exciton energy in cuboidal $CsPbBr_3$ QDs calculated by Blundell and Guet, Ref.10, using the particle-hole Bethe-Salpeter equation to that calculated using Eq. S13 but using the same parameters as Ref.10. The methods agree within the resolution of the plot digitization of the calculation in Ref.10. In Fig. S3b, we make a similar comparison of the calculated radiative lifetimes, discussed below. The comparison shows that the effect of the correlated electron/hole motion captured by the variational method reproduces the effects of correlation found in the all-order many-body approaches within the intermediate confinement size regime.

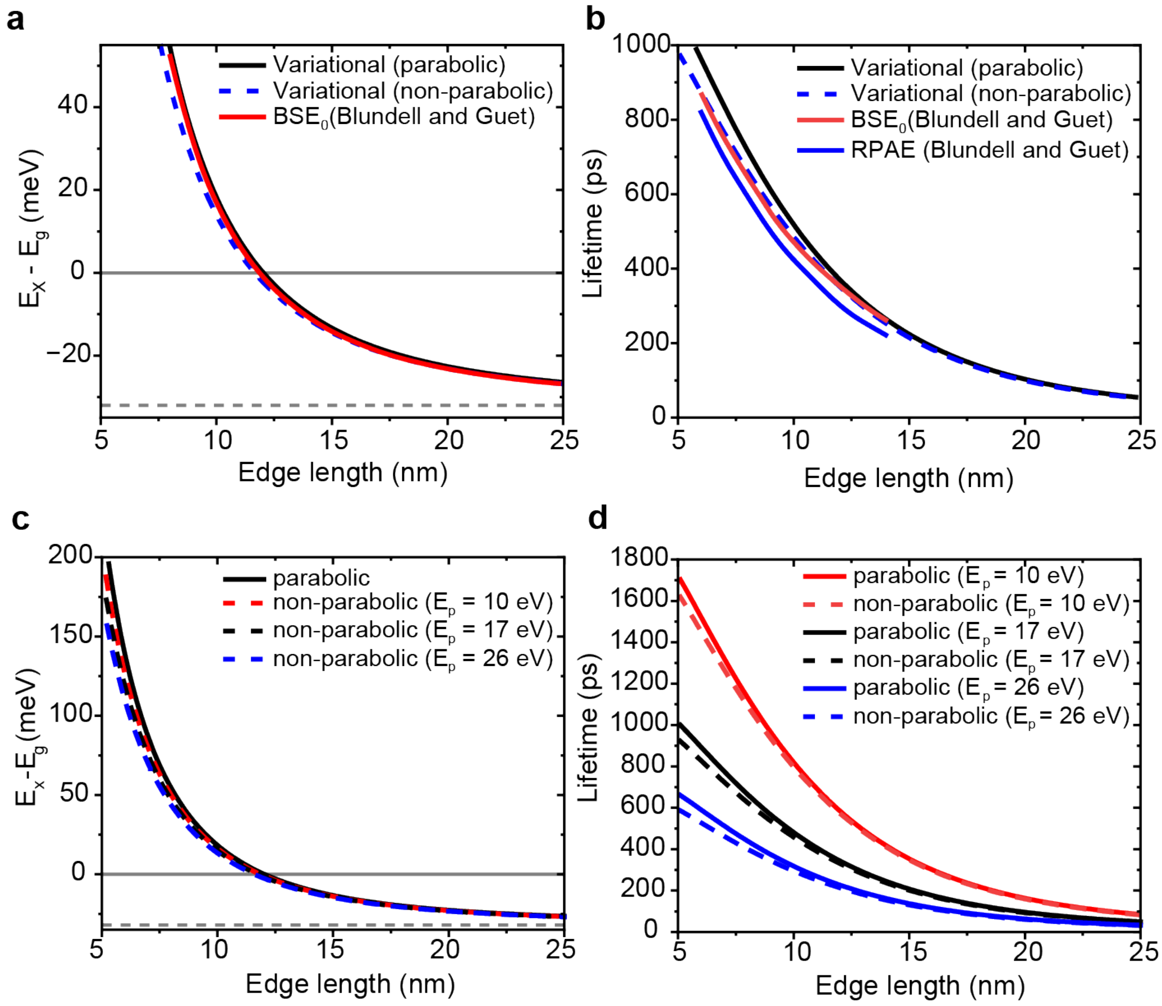

Fig. S3 | Comparison of exciton energy and radiative decay rate calculated variationally versus all-order many-body techniques applied within the effective-mass approximation; and examination of non-parabolicity effects. Panel **a** shows the exciton energy, E_x, relative to the bulk band gap, E_g, versus QD edge length, L, using the material parameters of Ref.10: $\epsilon_{eff} = 7.3$; $\epsilon_{NC,\infty} = 4.84$; $\epsilon_{med} = 2.4$; $E_p = 20\ eV$. The solid black line shows the variation calculation, Eq. S13, while the blue dashed line is the variational calculation with corrections for non-parabolicity (see Eq. S26). The solid red line is a plot of the calculated result reported by Blundell and Guet in Ref.10, using particle-hole Bethe-Salpeter equation (BSE_0). The BSE_0 and the variational lines are not meaningfully distinguishable. Panel **b** shows the corresponding comparison between the radiative lifetime calculated variationally (parabolic band approximation, solid black line), Eq. S23, and including non-parabolicity corrections, dashed blue line) with the results reported in Ref.10 using BSE ("BSE_0", solid red line) and the random-phase approximation with exchange including K.P corrections ("RPAE", solid blue line). Over the range for which the lifetime is reported in Ref.10, the variational calculation agrees within 10% with the results of the BSE_0 method. Panel **c** and **d** show the calculation of the exciton energy and radiative lifetime using parameters from Table S3 for Kane energies 10 eV, 17 eV, and 26.5 eV.

B. Low-temperature photoluminescence decay lifetime

Oscillator strength.

Within the intermediate confinement model developed above, the exciton oscillator strength is proportional to the square of the interband momentum matrix element and inversely proportional to the normalization integral $\mathcal{N}$. The average of the oscillator strength over the bright triplet exciton levels can be shown to be given by Ref.5,

$$\bar{f}(L) = \frac{2}{3}\frac{f_o}{\mathcal{N}(b_{opt}(L))} \ , \qquad \text{(S16)}$$

where $f_o = E_p/\hbar\omega$ is the ratio of E_p, the Kane energy, and the transition energy, $\hbar\omega$. The Kane energy is proportional to the square of the interband dipole matrix element; it is defined by the relation $E_p = 2\,|P|^2/m_0$, where $P = -i\,\langle s|\hat{p}|z\rangle$ is the Kane interband momentum matrix element, and m_0 is the free electron mass[11].

For comparison with the result found in the intermediate confinement regime, the average of the exciton oscillator strength in strong confinement is $\bar{f}_{sc} = 2/3\, f_o$, a result which reflects the absence of correlated electron-hole motion. The factor $1/\mathcal{N}(b_{opt}(L))$ thus represents an enhancement of the oscillator strength due to the correlated motion of the electron and hole. In the limiting case of weak confinement, realized in large QDs, the electron/hole motion is strongly correlated as reflected in hydrogenic relative electron/hole motion with quantum confinement of the center-of mass motion. In this limit the exciton envelope function is given by Ref.5,

$$f(\boldsymbol{r}_e, \boldsymbol{r}_h) = \varphi_{1s}(r)\,\psi_S(X,Y,Z)\,. \qquad \text{(S17)}$$

Here, $\varphi_{1s}(r)$ is the 1s hydrogenic wavefunction in the relative coordinate $r = |\boldsymbol{r}_e - \boldsymbol{r}_h|$, while X, Y, Z are the cartesian components of the center-of-mass coordinate $\boldsymbol{R}$, and ψ_S is given in Eq.

S3. This leads to the expression for the average oscillator strength of the bright triplet levels in a cuboidal QD of volume $V = L_x L_y L_z$ shown in Ref.12-14,

$$\bar{f}_{weak}(V) = \frac{2}{3} f_o \frac{512}{\pi^6} \left(\frac{V}{\pi a_x^3} \right), \tag{S18}$$

where a_x is the exciton Bohr radius. We see that the oscillator strength grows linearly with the QD volume in the limit of large size QDs with characteristic edge lengths $L \gg a_x$, a result anticipated by Rashba in his seminal work on the giant oscillator transition strength of impurity bound excitons[15]. To illustrate, we show in Fig. S4 a plot of the size dependence of the oscillator strength in the strong, intermediate and weak confinement approximations. As expected, the intermediate confinement result matches the strong confinement in the small size limit and transitions at large size to the linear scaling with nanocrystal volume characteristic of the large size limit where the weak confinement limit is realized.

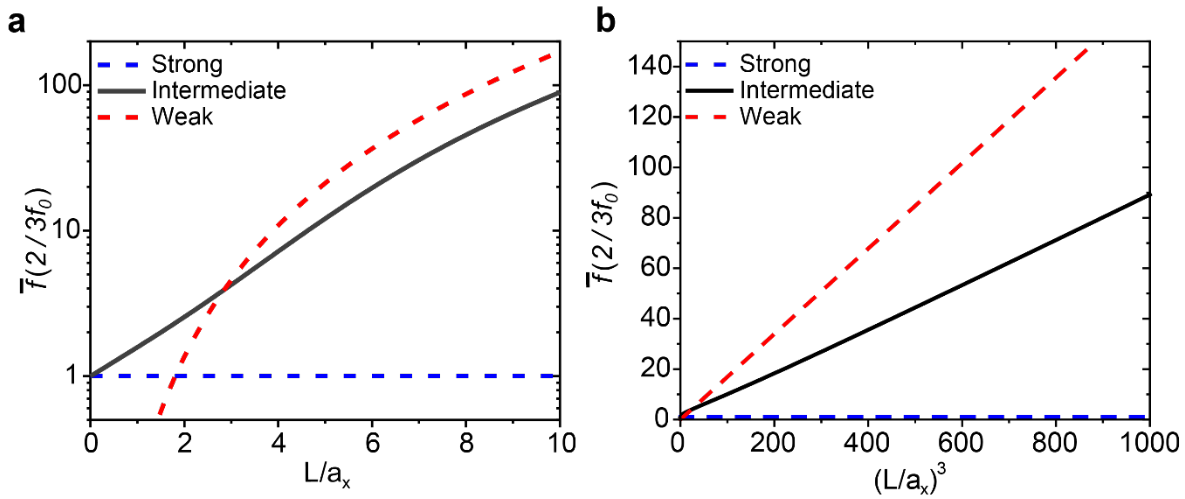

Fig. S4 | Average bright exciton oscillator strength versus QD size. a, The average bright exciton oscillator strength $\bar{f}$ in units of $^2/_3 f_o$ is plotted versus the ratio L/a_x of the edge length, L, of a cube-shaped QD to the exciton radius, a_x. The solid black line represents the result of the variation calculation Eq. S16. For reference the average oscillator strength calculated in the strong and weak confinement limit (blue dashed line, red dashed line, respectively) are shown as well. **b**, Plot of the average oscillator strength versus cube of the ratio L/a_x showing that in intermediate

confinement the oscillator strength goes over to the linear scaling with respect to volume in the large size limit, which is characteristic of the weak confinement limit.

Radiative lifetime.

The size dependence of the oscillator transition strength of QDs described above is reflected in a size dependent radiative decay rate. Within the intermediate confinement model, for each exciton state whose wavefunction is written in the form given in Eq. S4, the radiative lifetime is given by the expression[3]:

$$\left(\frac{1}{\tau(L)}\right) = \frac{4\,\omega\,n\,E_p}{9\cdot 137\,m_0\,c^2} D^2\ \frac{\left|\langle\psi_{S,e}|\psi_{S,h}\rangle\right|^2}{\mathcal{N}(b_{opt}(L))}. \tag{S19}$$

Here, n is the refractive index of the medium surrounding the QD, D is the dielectric depolarization factor, reflecting the ratio of the volume averaged electric field internal to the QD to the field amplitude outside the QD, c is the speed of light in vacuum, and the other terms have been previously defined. For spherical QDs, which well represent the studied large QDs (see Extended Data Fig. 1), and with high frequency dielectric constant internal to the QD, $\epsilon_{QD,\infty}$, embedded in a medium with dielectric constant ϵ_{med}, the factor $D = D_{sph}$ is given in terms of the ratio $\kappa = \epsilon_{QD,\infty}/\epsilon_{med}$ by[3],

$$D_{sph} = \frac{3}{2+\kappa}, \tag{S20}$$

$$\left(\frac{1}{\tau(L)}\right) = \frac{4\,\omega\,n\,E_p}{9\cdot 137\,m_0\,c^2}\ D_{sph}^2\ \frac{\left|\langle\psi_{S,e}|\psi_{S,h}\rangle\right|^2}{\mathcal{N}(b_{opt}(L))}. \tag{S21}$$

Eq. S21 can be simplified by noting that the overlap $\langle\psi_{S,e}|\psi_{S,h}\rangle$ ~1. Using this, we re-cast the expression in terms of the radiative lifetime of an uncorrelated e/h pair at the band edge, τ_0:

$$\tau_0 = \frac{9 \cdot 137\, m_0\, c^2}{4\; n\, E_p\; D_{sph}^2}\, \frac{\hbar}{E_g}\;; \tag{S22}$$

$$\left(\frac{1}{\tau(L)}\right) = \frac{1}{\tau_0}\left(\frac{E(L)}{E_g}\right)\; \frac{1}{\mathcal{N}\left(b_{opt}(L)\right)}. \tag{S23}$$

Eqs. S21-S23 show that apart from the giant oscillator strength enhancement factor $1/\mathcal{N}(b_{opt}(L))$ the radiative lifetime principally depends on a number of other parameters, notably, the value of the Kane energy, E_p. As above in our discussion of the exciton energy, it is instructive to compare these expressions to calculations made using more sophisticated many-body techniques. In Fig. S3b, we show a comparison between the radiative lifetime calculated variationally (solid black line), Eq. S21-S23, with the results shown in Ref.10 using BSE ("BSE_0", solid red line) and the random-phase approximation with exchange ("RPAE", solid blue line) using the parameters of Ref.10. Over the range for which the lifetime is reported in Ref.10, the variational calculation agrees with the results of the BSE method remarkably well, as expected given the near exact correspondence in the calculated energies shown in Fig. S3a. To proceed further in calculating the radiative lifetime it is necessary to review the materials parameters for $CsPbBr_3$.

Materials parameters for $CsPbBr_3$.

As discussed above, the exciton reduced effective mass and effective dielectric parameters were experimentally determined by Yang *et al.*, Ref.9, for bulk thin films of $CsPbBr_3$ by analysis of exciton magneto-transmission measurements at 2 K. These parameter values are used throughout this work and are shown in Table S2. The bulk PL bandgap was determined in this

work by a one parameter fit of the ensemble QD PL emission energy versus size at temperature 4 K shown in Fig. 2b of the main text, using the reduced effective mass and effective dielectric constant from Ref.9 and taking the bulk bandgap as a fitting parameter. The resulting bulk PL bandgap is determined as E_g =2361 meV as shown in Table S2. Other parameters required in the lifetime model are the high frequency dielectric constant inside the QD and in the surrounding polystyrene medium, whose numerical values are taken from the literature and shown in Table S2.

Critically, to evaluate the spontaneous emission lifetime we need the Kane energy, E_p, for $CsPbBr_3$, which is not a directly measured parameter. As noted above, the Kane energy is given by $E_p = 2\,|P|^2/m_0$, where $P = -i\,\langle s|\hat{p}|z\rangle$ is the Kane inter-band momentum matrix element, and m_0 is the free electron mass[11]. Its value can be estimated from the reduced effective mass of the exciton, which has been measured in thin-film $CsPbBr_3$[9]. However, this procedure is uncertain since the effective mass depends not only on the Kane energy but on several additional parameters whose values are either unknown or not well known: these are the spin-orbit split-off parameter, Δ, which separates the upper ($j = 3/2$) conduction bands from the lowest ($j = 1/2$) conduction bands, and terms representing remote band contributions to the effective mass.

Table S2. Materials parameters for $CsPbBr_3$ and derived model parameters.

Parameter	Value	Source
Exciton reduced effective mass μ/m_0	0.126	Ref. 9
Exciton effective dielectric constant ϵ_{eff}	7.3	Ref. 9
PL bulk band gap, E_g	2361 meV	This work, fit to sizing curve in Fig. 2b.
Exciton Bohr radius in bulk $CsPbBr_3$, a_x	3.07 nm	$a_x = a_o \epsilon_{eff}/\mu$ where a_o is the hydrogen Bohr radius
Exciton binding energy in bulk $CsPbBr_3$, B_x	32 meV	$B_x = \dfrac{\hbar^2}{2\mu a_x^2}$
$CsPbBr_3$ high frequency dielectric constant $\epsilon_{NC,\infty}$	4.3	Ref. 16
Polystyrene dielectric $\epsilon_{med} = n^2$ at 525 nm	2.56	Ref. 17
Dielectric screening factor D_{sph} ($\kappa = 1.7$)	0.815	Eq. S20

In Refs.6,7, the Kane energy was estimated as 26.5 eV and 28 eV, respectively, from effective mass theory within a 4-band Kane model. However, the 4-band model is only valid in the limit that the spin-orbit split-off parameter, Δ, is large relative to the band gap; in fact, this limit is not realized in $CsPbBr_3$, resulting in an overestimation of the Kane energy using this procedure[10,18]. Proper inclusion of coupling involving the upper $j = 3/2$ conduction bands and inclusion of remote band contributions results in the following expressions for the band edge electron and hole effective masses derived in an 8-band K.P model[19]:

$$\frac{m_0}{m_e} = \gamma_1 + \frac{1}{3}\frac{E_p}{E_g}\,; \qquad \frac{m_0}{m_h} = \alpha + \frac{E_p}{3}\left(\frac{1}{E_g} + \frac{2}{E_g + \Delta}\right) \tag{S24}$$

In these expressions, E_g is the bulk band gap, E_p is the Kane energy, m_0 is the free electron mass; while γ_1 and α represent remote band contributions to the effective masses. From the expressions above we arrive at the band edge exciton reduced effective mass: Using $A = \gamma_1 + \alpha$,

$$\frac{m_0}{\mu} = A + \frac{2\,E_p}{3}\left(\frac{1}{E_g} + \frac{1}{E_g + \Delta}\right) \tag{S25}$$

Inclusion of the band non-parabolicity which occurs within an 8-band K.P model can be accomplished by replacing the band gap E_g by the QD band gap, $E_g \rightarrow E_g + \Delta E_{conf}$[20]:

$$\frac{m_0}{\mu(E)} = A + \frac{2\,E_p}{3}\left(\frac{1}{E} + \frac{1}{E + \Delta}\right)\ ;\quad E = E_g + \Delta E_{conf}\ ; \tag{S26}$$

and iterating to convergence. For larger QD sizes within range studied here, we found that the effect of non-parabolicity is small. To illustrate the effect, in Fig. S3 we compare the calculated exciton energy and radiative lifetime found within a simple parabolic model against the result accounting for the band non-parabolicity for the parameters of Ref.10 in panels a and b; and in panels c and d for parameters chosen from Table S3 spanning the low to high range of the Kane energy. Inspection of the figure shows that for larger values of the Kane parameter the non-parabolicity effect is enhanced as expected.

It is important to note that due to uncertainty in the parameters Δ and the remote band term A, it is not possible to definitively establish the value of the Kane energy solely from the exciton reduced effective mass. This is shown in Table S3, which shows the value of the Kane energy derived from the measured exciton reduced effective mass reported in Ref.9 but using different values of the split-off parameter Δ taken from the literature with compensating values of the remote band mass parameter A to preserve the measured reduced effective mass. Estimates for the Kane energy range from 10-26.5 eV. Also shown in the table is the band-edge uncorrelated e/h pair lifetime, τ_0, resulting from the calculated Kane energy for each case.

An alternative constraint is provided by the dependence of the hole Landé g-factor on the Kane energy. The hole g-factor has recently been measured as g_h= + 0.83 in $CsPbBr_3$ QDs as reported by Grigoryev *et al.* in Ref.21. Within an 8-band K.P model[22], the hole g-factor for the cubic phase (which is approximately equal to the orientational average for the orthorhombic phase) is determined by the Kane energy and the split-off parameter, Δ (in addition to the bandgap, which is readily measured):

$$g_h = g_0 - \frac{2}{3}E_p\left(\frac{1}{E_g} - \frac{1}{E_g + \Delta}\right) \tag{S27}$$

Reported estimates of Δ inferred from optical absorption spectra range from 0.8-1.2 eV[10,23], while hybrid density functional theory calculations give a higher value Δ = 1.5 eV[6]. The uncertainty in Δ affects the estimates of the Kane energy, which consequently range from 10.2 eV (for Δ = 1.5 eV) to 16 eV (for Δ = 0.8 eV) based on the g-factor measurement of Ref.21 as shown in Table S3.

To show the impact of the Kane energy on the lifetime calculation, we plot the lifetime utilizing different Kane energy values (E_p) in Fig. 2d. The values of 10 eV, 12 eV and 16 eV are all obtained from the measured hole g-factor[21] using an 8-band k.p model, respectively using Δ = 1.5 eV, determined using hybrid density functional theory[6], Δ = 1.2 eV from low temperature absorption spectra of $CsPbBr_3$ QDs as assigned by Ramade *et al.* in Ref.23 and Δ = 0.8 eV, also determined from absorption spectra but as assigned by Blundell *et al.* in Ref.10. The value E_p = 17.5 eV was determined from the exciton reduced effective mass[9] within an 8-band K.P model with no remote band terms using Δ = 1.5 eV, while the value E_p = 26.5 eV was originally determined from the exciton reduced effective mass[9] within a 4-band K.P model[7] but can be rationalized within an 8-band K.P model by inclusion of remote band mass term as shown in Table

S3. The value E_p = 20.0 eV was proposed by Blundell *et al.* in Ref.10 as an intermediate value between the 4-band and the 8-band result.

With decreasing E_p, the radiative lifetime shortens for any given QD size, indicating the impact of E_p. For all of the theoretical curves, the exciton lifetime reduces with increasing QD size, qualitatively capturing the experimental trend in single-QD lifetimes shown in the main text. Overall, the shortening of lifetime with increasing QD size is consistent with exciton wavefunction delocalization in larger QDs, a typical feature of super-fluorescent systems generally and the exciton giant oscillator transition strength effect in particular.

Among all the theoretical calculations, the theory with $E_p = 16 - 17$ eV shows the best agreement with the measured data shown in Fig. 2d.

Table S3. Estimates for Kane energy in $CsPbBr_3$.

The table shows estimates of the Kane energy consistent with the reduced effective mass μ/m_0 = 0.126 reported in Ref.9 for different values of the split-off parameter Δ taken from the literature based either on hybrid DFT or absorption ("Abs."). The bulk bandgap E_g = 2361 meV was determined by fitting the QD PL emission energy versus size shown in Fig. 2b. The hole g-factor is calculated at exciton energy 2330 meV for comparison to the measurement reported in Ref.21. Other parameters used in calculating the band-edge uncorrelated pair lifetime τ_0 in the table are given in Table S2.

Split-off parameter	Source	Remote band mass term A	Kane energy E_p	Hole g-factor g_h	Band-edge uncorrelated pair lifetime τ_0
1.543 eV	Hybrid DFT[6]	3.28	10.28 eV	0.830	4015 ps
1.2 eV	Abs[23]	2.28	12.05 eV	0.830	3426 ps
0.8 eV	Abs[10]	0.029	16.03 eV	0.830	2576 ps
0.8 eV	Abs[10]	0	16.09 eV	0.826	2566 ps
1.543 eV	Hybrid DFT[6]	0	17.51 eV	0.006	2357 ps
0.8 eV	Abs[10]	-1.93	20 eV[10]	0.540	2065 ps
1.543 eV	Hybrid DFT[6]	-4.08	26.5 eV	-1.018	1558 ps

C. Connection to Dicke's theory of superradiance

In the preceding section we have shown how the structure of the exciton envelope wavefunction in the intermediate confinement regime leads to giant oscillator strength, scaling linearly with the nanocrystal volume for large nanocrystal size $L \gg a_x$, which gives rise to accelerated radiative decay. Here we establish the connection between the giant oscillator strength, the resulting accelerated decay, and single photon superradiance in small N atom clouds, as described in the seminal work of Dicke[24].

In the case of Dicke superradiance, a symmetric single photon excitation of an N atom cloud will decay at a rate faster by the factor N than a single isolated atom; this acceleration is due to an increase in the transition dipole moment for decay or absorption by the factor $\sqrt{N}$. As has previously been noted[13,14,25], the fast decay in large sized quantum dots occurs due to the fact that the transition dipole moment of the lowest energy exciton energy level (apart from spin) is coherent over the entire nanocrystal quantum dot; this coherency leads to a giant transition dipole moment, which is a hallmark of superradiance.

The single photon N-atom Dicke state.

To see the connection to Dicke's theory of single photon superradiance we will make an argument similar the one advanced by Tighineanu *et al.*[25]. We begin by writing the symmetric single-photon N-atom Dicke state, where the N-atom cloud has a size $R \ll \lambda$ where λ is the wavelength of light[26]:

$$\Psi_N = \frac{1}{\sqrt{N}} \sum_j \varphi_e(j) \prod_{i \neq j} \varphi_g(i) \, . \tag{S28}$$

Here, the indices j, i run over the N atoms, while the terms $\varphi_e(j)$ and $\varphi_g(j)$ denote the state of a given atom, j, in its excited or its ground state, respectively. In this notation the ground state of the system is,

$$G_N = \prod_i \varphi_g(i) \,. \tag{S29}$$

It is straightforward to show using these wavefunctions that the transition dipole matrix element between the ground state and the symmetric single photon Dicke state scales as $|p| = \langle \Psi_N | \hat{p} | \, G_N \rangle \sim \sqrt{N}$, and that therefore the oscillator strength and thus the radiative emission rate scale as $f \sim N$, the number of atoms.

The exciton state in intermediate confinement.

Now for comparison to the N-atom single photon Dicke state, we consider the exciton wavefunction in a QD in the intermediate confinement regime. The equation for the exciton envelope function was given previously in Eq. S4. The total exciton wavefunction, $\Psi_{exc}(\boldsymbol{r}_e, \boldsymbol{r}_h)$, is the product of the envelope function $f(\boldsymbol{r}_e, \boldsymbol{r}_h)$ with the band-edge cell-periodic Bloch functions for the electron and hole, $u_e(\boldsymbol{r}_e)$ and $u_h(\boldsymbol{r}_h)$, respectively. These have the property that $u(\boldsymbol{r} + \boldsymbol{T}) = u(\boldsymbol{r})$ where $\boldsymbol{T}$ is a Bravais lattice vector of the semiconductor. The normalization of these functions is,

$$\int_{\Omega} u_m^*(\boldsymbol{r}) \, u_n(\boldsymbol{r}) \, d^3r = \Omega \, \delta_{n.m} \,, \tag{S30}$$

where Ω is the volume of a unit cell and $\delta_{n.m}$ is the Kronecker delta. Using the band edge Bloch functions for the electron and hole the total wave function in intermediate confinement is,

$$\Psi_{exc}(\boldsymbol{r}_e, \boldsymbol{r}_h) = \frac{1}{\sqrt{\mathcal{N}(\beta)}} e^{-\beta|r_e - r_h|} \psi_S(\boldsymbol{r}_e)\psi_S(\boldsymbol{r}_h)\, u_e(\boldsymbol{r}_e)\, u_h(\boldsymbol{r}_h). \tag{S31}$$

To see the connection to the Dicke's state, Eq. S28, we convert this expression from a band-edge Bloch function basis to a basis of lattice-site localized Wannier functions. The required transformation is derived using the definition of the Wannier function in terms of the delocalized Bloch functions, $\psi_{n,\boldsymbol{k}}(\boldsymbol{r})$, for band n and wave vector $\boldsymbol{k}$, in a semiconductor crystal comprised of N unit cells. The Wannier function localized at lattice site $\boldsymbol{T_j}$ is given by,

$$w_n(\boldsymbol{r} - \boldsymbol{T_j}) = \frac{1}{\sqrt{N}} \sum_{\boldsymbol{k}} e^{-i\,\boldsymbol{k}\cdot\boldsymbol{T}_j}\, \psi_{n,\boldsymbol{k}}(\boldsymbol{r}), \tag{S32}$$

where the sum is over the N discrete wave vectors $\boldsymbol{k}$ in a crystal comprised of N unit cells. The inverse transformation is,

$$\psi_{n,\boldsymbol{k}}(\boldsymbol{r}) = \frac{1}{\sqrt{N}} \sum_{j} e^{+i\,\boldsymbol{k}\cdot\boldsymbol{T}_j}\, w_n(\boldsymbol{r} - \boldsymbol{T_j}). \tag{S33}$$

where the sum is over the N unit cells each located at Bravais lattice vector $\boldsymbol{T_j}$ comprising the crystal. The Bloch functions for band n and wave vector k, $\psi_{n,\boldsymbol{k}}(\boldsymbol{r})$ are also given by Bloch's theorem,

$$\psi_{n,\boldsymbol{k}}(\boldsymbol{r}) = \frac{1}{\sqrt{V}} e^{i\,\boldsymbol{k}\cdot\boldsymbol{r}} u_{n,\boldsymbol{k}}(\boldsymbol{r})\,. \tag{S34}$$

For near band-edge states we approximate $u_{n,\boldsymbol{k}}(\boldsymbol{r}) \cong u_{n,\boldsymbol{0}}(\boldsymbol{r}) \equiv u_n(\boldsymbol{r})$. Using this with Eq. S33-S34 we find,

$$u_n(\boldsymbol{r}) \cong \sqrt{\Omega} \sum_{j} w_n(\boldsymbol{r} - \boldsymbol{T_j})\,. \tag{S35}$$

Using this in Eq. S31 and using the fact that the envelope functions are slowly varying on the scale of a unit cell, we find the "Wannierized" exciton wavefunction:

$$\Psi_{exc}(\boldsymbol{r}_e, \boldsymbol{r}_h) = \tag{S36}$$

$$\frac{1}{\sqrt{\mathcal{N}(\beta)}} \sum_j \sum_{j'} e^{-\beta\left|\boldsymbol{T}_j - \boldsymbol{T}_{j'}\right|} \tilde{\psi}_S(\boldsymbol{T}_j) \tilde{\psi}_S(\boldsymbol{T}_{j'})\, w_e\,(\boldsymbol{r}_e - \boldsymbol{T}_j)\, w_h\,(\boldsymbol{r}_h - \boldsymbol{T}_{j'})\,,$$

where $\tilde{\psi}_S(\boldsymbol{T}_j)$ is the discretized form of the envelope function $\psi_S(\boldsymbol{r})$, constructed according to the transformation, $\tilde{\psi}_S(\boldsymbol{T}_j) = \sqrt{\Omega}\, \psi_S(\boldsymbol{r})\, \delta_{\boldsymbol{r},\boldsymbol{T}_j}$, so that the sum $\sum_j \left|\tilde{\psi}_S(\boldsymbol{T}_j)\right|^2 = 1$, given that the function $\psi_S(\boldsymbol{r})$ is normalized over the nanocrystal volume (see Eq. S8).

The Wannier site-localized form of the exciton wavefunction, Eq. S36, is written in the electron/hole representation. To contact the single photon superradiant state it is useful to express this in the electron representation. This is accomplished by noting that the hole Wannier function $w_h\,(\boldsymbol{r} - \boldsymbol{T}_{j'})$ represents the absence of an electron in the valence band Wannier state $w_v\,(\boldsymbol{r} - \boldsymbol{T}_{j'})$; all other valence band Wannier functions are occupied. Thus,

$$\Psi_{exc}^{ee}(\boldsymbol{r}, \boldsymbol{r}') = \tag{S37}$$

$$\frac{1}{\sqrt{\mathcal{N}(\beta)}} \sum_j \sum_{j'} e^{-\beta\left|\boldsymbol{T}_j - \boldsymbol{T}_{j'}\right|} \tilde{\psi}_S(\boldsymbol{T}_j) \tilde{\psi}_S(\boldsymbol{T}_{j'})\, w_e\,(\boldsymbol{r} - \boldsymbol{T}_j) \prod_{i \neq j'} w_v\,(\boldsymbol{r}' - \boldsymbol{T}_i)\,,$$

where we ignore spin in the interests of simplicity of notation. In the same picture, the ground state of the crystal with N unit cells is represented as,

$$G_N = \prod_i w_v\,(\boldsymbol{r} - \boldsymbol{T}_i)\,. \tag{S38}$$

The analogy between Eq. S38 and the ground state in the N-atom cloud case, Eq. S29, is obvious: The role of the atomic ground state for atom i, $\varphi_g(i)$, in the Dicke state is filled here by the Wannier site function $w_v\,(\boldsymbol{r} - \boldsymbol{T}_i)$**.** To see the connection between the exciton state, Eq. S37 and the symmetric N-atom single photon Dicke state, Eq. S28, it is instructive to examine the exciton wavefunction, Eq. S37, in the limit of large correlation parameter, β. In this limit, the

correlation term $e^{-\beta|\boldsymbol{T}_j-\boldsymbol{T}_{j'}|}$ vanishes unless $\boldsymbol{T_j} = \boldsymbol{T}_{j'}$. In that case, the exciton wavefunction becomes,

$$\Psi_{exc}^{ee}(\boldsymbol{r},\boldsymbol{r}') \ \overrightarrow{\beta \to \infty}$$

$$\frac{1}{\sqrt{\mathcal{N}(\beta)}}\sum_j \tilde{\psi}_S(\boldsymbol{T}_j)\tilde{\psi}_S(\boldsymbol{T}_j)\, w_e\,(\boldsymbol{r}-\boldsymbol{T_j}) \prod_{i\neq j'} w_v\,(\boldsymbol{r}'-\boldsymbol{T_i})\,. \tag{S39}$$

This expression, which corresponds to the Frenkel exciton limit, is analogous to the N-atom single photon Dicke state (cf. Eq. S28) apart from an amplitude weighting function $\tilde{\psi}_S(\boldsymbol{T}_j)\tilde{\psi}_S(\boldsymbol{T}_j)$. Since the ψ_S are real valued functions, this state is a symmetric one like the symmetric single photon N-atom Dicke state. Not surprisingly, if the dipole transition matrix element is evaluated, we find,

$$\langle\Psi_{exc}^{ee}|\hat{p}|\, G_N\rangle \sim 1/\sqrt{\mathcal{N}(\beta)}\, p_o\,, \tag{S40}$$

where $p_o = \int_\Omega w_e{}^*(\boldsymbol{r})\, \hat{\boldsymbol{p}}\, w_v(\boldsymbol{r}) d^3r$. Since we have previously shown that for the large size limit, $b = \beta\, L_{eff} \gg 1$, the normalization integral $\mathcal{N}(b) \to (a_{x/}L_{eff})^3$, it is clear that the transition dipole scales as the square root of the nanocrystal volume, and that the oscillator strength scales as the volume, or equivalently, the number of unit cells, just as in the N-atom single photon Dicke state, and for the same reason.

Strong confinement limit.

It is equally instructive to examine the exciton wavefunction, Eq. S37, in the small size limit, $b = \beta\, L_{eff} \ll 1$. In this limit, the exponential term becomes unity, the normalization integral $\mathcal{N}(b) \to 1$, and we have a completely uncorrelated exciton as expected for the strong confinement limit:

$$\Psi_{exc}^{ee}(\boldsymbol{r},\boldsymbol{r}') \ \overrightarrow{\beta \to 0} \tag{S41}$$

$$\frac{1}{\sqrt{\mathcal{N}(\beta)}}\sum_{j}\sum_{j'}\tilde{\psi}_S(\boldsymbol{T}_j)\tilde{\psi}_S(\boldsymbol{T}_{j'})\, w_e\,(\boldsymbol{r}-\boldsymbol{T_j})\,\prod_{i\neq j'} w_v\,(\boldsymbol{r}'-\boldsymbol{T_i}).$$

This state is not a Dicke state; there is no correlation. Using this wavefunction $\langle\Psi_{exc}^{ee}|\hat{p}|\,G_N\rangle \sim p_o$ and consequently there is no scaling of the transition dipole or the oscillator strength with nanocrystal volume (as expected). This is not surprising since the giant oscillator transition strength is a consequence of the correlation between the electron and the hole.

Weak confinement limit.

To recapitulate the discussion so far: We have shown that in the strong confinement limit, $\beta \to 0$, where there is no electron/hole correlation, the exciton wavefunction is not a Dicke state and there is no oscillator strength enhancement. Conversely, in the limit of large correlation $\beta \to \infty$ the exciton wavefunction is a Dicke state, leading to the sought enhancement. However, this limit is artificial in that it formally corresponds to the Frenkel exciton limit as noted above. What is the character of the state in the more realistic limit of the Wannier exciton, for which in the large size limit, the correlation parameter goes to the inverse of the bulk exciton radius, $\beta \to 1/a_x$, so that the exciton wavefunction goes over to the weak confinement limit?

In the weak confinement limit, the total exciton wavefunction can be written,

$$\Psi_{exc}(\boldsymbol{r}_e,\boldsymbol{r}_h) = \psi_S(\boldsymbol{R})\varphi_{1S}(r)\, u_e(\boldsymbol{r}_e)\, u_h(\boldsymbol{r}_h), \qquad \text{(S42)}$$

where, as in Eq. S17, $\boldsymbol{R} = (m_e\boldsymbol{r}_e + m_h\boldsymbol{r}_h)/(m_e+m_h)$ denotes the COM coordinate of the exciton; $\boldsymbol{r} = \boldsymbol{r}_e - \boldsymbol{r}_h$ denotes the coordinate of relative electron/hole motion; and the band edge Bloch functions are as employed in Eq. S37. We first analyze this expression by transforming the COM portion of the exciton wave function into the Wannier site representation. This analysis proceeds as follows: We rewrite the Bloch function portion explicitly in terms of the COM coordinate,

$$\Psi_{exc}(\boldsymbol{R},\boldsymbol{r}) = \psi_S(\boldsymbol{R})\varphi_{1S}(r)\, u_{e,h}(\boldsymbol{R},\mathbf{r})\,, \tag{S43}$$

Noting that the function $u_{e,h}(\boldsymbol{R},\mathbf{r}) \;=\; u_{e,h}(\boldsymbol{R}+\boldsymbol{T},\mathbf{r})$ is periodic in the Bravais lattice vectors, $\boldsymbol{T}$. In this form, we can recast the exciton wavefunction in a Wannier site basis in the COM coordinate $\boldsymbol{R}$. The Wannier function we need can be found from the bulk exciton wavefunction,

$$\Psi_{exc,\boldsymbol{K}}(\boldsymbol{R},\boldsymbol{r}) = \frac{e^{i\,\boldsymbol{K}\cdot\boldsymbol{R}}}{\sqrt{V}}\varphi_{1S}(r)\, u_{e,h}(\boldsymbol{R},\boldsymbol{r})\,, \tag{S44}$$

by using the Wannier transform, Eq. S32, and its inverse, Eq. S33, as applied to the exciton:

$$W_{exc}\big(\boldsymbol{R}-\boldsymbol{T_j},\boldsymbol{r}\big) = \frac{1}{\sqrt{N}}\sum_{K} e^{-i\,\boldsymbol{K}\cdot\boldsymbol{T}_j}\; \Psi_{exc,\boldsymbol{K}}(\boldsymbol{R},\boldsymbol{r}); \tag{S45}$$

$$\Psi_{exc,\boldsymbol{K}}(\boldsymbol{R},\boldsymbol{r}) = \frac{1}{\sqrt{N}}\sum_{j} e^{+i\,\boldsymbol{K}\cdot\boldsymbol{T}_j}\; W_{exc}\big(\boldsymbol{R}-\boldsymbol{T_j},\boldsymbol{r}\big) \tag{S46}$$

We find the Wannier function by combining Eq. S44 at $\boldsymbol{K} = 0$,

$\Psi_{exc,\boldsymbol{0}}(\boldsymbol{R},\boldsymbol{r}) = \frac{1}{\sqrt{V}}\varphi_{1S}(r)\, u_{e,h}(\boldsymbol{R},\boldsymbol{r})$, with Eq. S45-S46. The resulting relations are,

$$\varphi_{1S}(r)\, u_{e,h}(\boldsymbol{R},\boldsymbol{r}) \cong \sqrt{\Omega}\sum_{j} W_{exc}\big(\boldsymbol{R}-\boldsymbol{T_j},\boldsymbol{r}\big)\,. \tag{S47}$$

$$W_{exc}\big(\boldsymbol{R}-\boldsymbol{T_j},\boldsymbol{r}\big) \;\cong\; \frac{1}{\sqrt{\Omega}}\varphi_{1S}(r)\, u_{e,h}(\boldsymbol{R},\boldsymbol{r})\,\delta_{\boldsymbol{R},\boldsymbol{T}_j}\;.$$

Using these, we can write the confined exciton wavefunction in the Wannier representation:

$$\Psi_{exc}(\boldsymbol{R},\boldsymbol{r}) = \sum_{j}\tilde{\psi}_S\big(\boldsymbol{T_j}\big)W_{exc}\big(\boldsymbol{R}-\boldsymbol{T_j},\boldsymbol{r}\big)\,, \tag{S48}$$

where we have used the discretization, $\tilde{\psi}_S\big(\boldsymbol{T}_j\big) \;=\; \sqrt{\Omega}\;\psi_S(\boldsymbol{R})\,\delta_{\boldsymbol{R},\boldsymbol{T}_j}$. This form matches a similar expression in Ref.25 which was derived by convolution methods. Its straightforward to show using these expressions and $\varphi_{1S}(0) \;=\; 1/\sqrt{\pi a_x^3}$ that the transition dipole matrix element is given by,

$$\langle \Psi_{exc} | \hat{p} | G \rangle = \sqrt{N} \sqrt{\frac{\Omega}{\pi a_x^3} \frac{16\sqrt{2}}{\pi^3}} \, p_o \, , \tag{S49}$$

which scales as the square root of N, the number of unit cells in the nanocrystal, and leads directly to the expression Eq. S18 for the oscillator strength.

An alternate formulation of the Wannier site representation of the weak confinement wavefunction can be found from Eq. S42 by transforming both the electron and hole Bloch functions into the Wannier site representation using Eq. S35. In the electron/hole representation the result is,

$$\Psi_{exc}(\boldsymbol{r}_e, \boldsymbol{r}_h) = \sum_j \sum_{j'} \tilde{\psi}_S(\boldsymbol{R}_{j,j'}) \tilde{\phi}_{1S}(\boldsymbol{r}_{j,j'}) \, w_e\,(\boldsymbol{r} - \boldsymbol{T}_j)\, w_h\,(\boldsymbol{r} - \boldsymbol{T}_{j'}) \, , \tag{S50}$$

where $\boldsymbol{R}_{j,j'} = (m_e \boldsymbol{T}_j + m_h \boldsymbol{T}_{j'})/(m_e + m_h)$; $\boldsymbol{r}_{j,j'} = \boldsymbol{T}_j - \boldsymbol{T}_{j'}$; and where $\tilde{\psi}_S(\boldsymbol{R}_{j,j'}) = \sqrt{\Omega}\, \psi_S(\boldsymbol{R})\, \delta_{\boldsymbol{R},\boldsymbol{R}_{j,j'}}$ and $\tilde{\phi}_{1S}(\boldsymbol{r}_{j,j'}) = \sqrt{\Omega}\, \phi_{1S}(\boldsymbol{r})\, \delta_{\boldsymbol{r},\boldsymbol{r}_{j,j'}}$. This can be converted as before into the electron representation, leading to,

$$\Psi_{exc}^{ee}(\boldsymbol{r}, \boldsymbol{r}') = \sum_j \sum_{j'} \tilde{\psi}_S(\boldsymbol{R}_{j,j'}) \tilde{\phi}_{1S}(\boldsymbol{r}_{j,j'}) \, w_e\,(\boldsymbol{r} - \boldsymbol{T}_j) \prod_{i \neq j'} w_v\,(\boldsymbol{r}' - \boldsymbol{T}_i) \, . \tag{S51}$$

This expression matches the one derived for weak confinement in the site representation by Hanamura[13] and leads, with Eq. S38 for the ground state, to the transition dipole matrix element given by Eq. S49. Eq. S51 is similar in structure to Eq. S37 for intermediate confinement. As in the analysis of that case we see that in the limit of small exciton radius, $\tilde{\phi}_{1S}(\boldsymbol{r}_{j,j'})$ vanishes unless $\boldsymbol{T}_j = \boldsymbol{T}_{j'}$ leading again to an expression that, like Eq. S39, corresponds closely to an N-atom single photon Dicke state. Conversely for large exciton radius Eq. S50 goes over to an uncorrelated state

which exhibits no collective oscillator strength enhancement. In their general forms, Eqs. S40 and S50 represent generalizations of the N-atom single photon superradiant state described by Dicke as claimed by Tighineanu *et al*[25].

III. *Ab-initio* molecular dynamics simulations

We perform ab-initio molecular dynamics (AIMD) simulation at the Density functional theory (DFT) level framework based on previously reported geometry: optimized $CsPbBr_3$ and $CsPbBr_3/CsCaBr_3$ QD models[27] in vacuum (at least 1 nm on each side). First, our models comply with the experimentally found AX-type surface termination; second, to strike a well-proven balance between the employed level of theory and the importance of modelling realistic QD sizes, we opted for modelling computationally large QDs (up to about 4 nm in size), at the PBE level of theory without spin-orbit coupling. Such a choice has previously proven to reproduce a wide range of experimental observations, i.e. emission line broadening[27] to ligand chemistry[28].

Electronic structure calculations and molecular dynamics (MD) simulations were performed in CP2K[29] utilizing the Quickstep module with Gaussian and plane waves featuring a plane-wave cut-off of 300 Ry[30]. We employed DZVP-MOLOPT basis sets, Goedecker-Teter-Hutter pseudopotentials[31-33] and Perdew-Burker-Enzerhof exchange-correlation functionals[34].

MD simulations were performed within the NVT ensemble employing a CSVR thermostat[35] with a time-constant of 15 fs at the respective temperatures or in the NVE ensemble after thermal equilibration. 20 ps long MD trajectories were simulated with 1 or 10 fs timesteps and at least the first 6 ps were considered as equilibration. After equilibration, we quantify the localization of the HOMO wavefunction every 0.1 ps via the associated probability density (.cube files).

To this end, we modelled the temperature-dependent electronic structure in realistically sized perovskite QDs *via ab-initio* molecular dynamics (AIMD) at the DFT level of theory. Our main results are presented in Extended Data Fig. 3 and 4. In Extended Data Fig. 3a, we first highlight that the spatial extension of the wavefunction strongly increases with QD size, in qualitative agreement with the increased coherence volume in our k.p analysis and the experimentally observed speedup of the radiative decay. With increasing temperature, however, our experiments had suggested a progressive loss of such superradiant state. Here, we provide mechanistic insights into the origin of this temperature dependence by revealing a pronounced wavefunction localization associated with phonon-induced electronic disorder. Visualizing the wavefunction localization across our experimentally studied temperature range, Extended Data Fig. 3b displays representative molecular dynamic (MD) snapshots of the HOMO density distribution both at cryogenic temperature (3 K, left image) and increased temperature (150 K, right image). Extended Data Fig. 3c quantifies the thermally induced wavefunction localization also for several intermediate temperatures, expressed via the time-averaged inverse participation ratio (IPR) of the associated probability density $\rho(r) = |\Psi(r)|^2$

$$IPR = \frac{\int \rho(r)^2 dr}{\left(\int \rho(r) dr\right)^2}, \quad \text{(S52)}$$

which increases for increasing localization[36]. Phonon induced fluctuations in the position of the HOMO wavefunction are captured by the time-averaged root-mean-squared displacement (RMSD) of the center of the HOMO density from the QD center (Extended Data Fig. 3d). We exclude contributions of the thermostat of the simulations in the canonical ensemble (NVT, blue line) by validating our analysis with MD trajectories obtained in the microcanonical ensemble (NVE, red points). Having established the equivalence, in the following we present an analysis based on AIMD simulations in the canonical ensemble only. In addition, we have included a movie

containing snapshots of the wavefunction, which can help to visually transmit the rich data set obtained from the AIMD simulations as other supplementary materials. All highlight the importance of minimizing the coupling to phonons to allow wavefunction delocalization and attaining single-photon superradiance.

Beyond the spatial visualizations, we here also provide a detailed analysis of how thermally activated phonons induce dephasing, and, thus, loss of coherence. Extended Data Figs. 3e, 3f display for the HOMO time-dependent dephasing function $D(t,T)$ at T=3 K and T=150 K, respectively, as obtained from the optical response formalism[37]

$$D(t,T) = e^{-g(t,T)}, \tag{S53}$$

where $g(t,T)$ is the lineshape function

$$g(t,T) = \frac{1}{\hbar^2}\int_0^t d\tau_1 \int_0^{\tau_1} d\tau_2 C(\tau_2,T) \tag{S54}$$

which is obtained from $C(\tau_2,T) = \langle \Delta E_{HOMO}(\tau_2,T)\Delta E_{HOMO}(0,T)\rangle$, the autocorrelation function of the HOMO energy fluctuation $\Delta E_{HOMO}(\tau_2,T) = E_{HOMO}(\tau_2,T) - \langle E_{HOMO}(\tau_2,T)\rangle$. Square brackets $\langle\ldots\rangle$ denote time-averaging, and the underlying HOMO energy fluctuations $E_{HOMO}(\tau_2, T = 3\ K)$ and $E_{HOMO}(\tau_2, T = 150\ K)$ from our AIMD simulations are shown in the inset of Extended Data Figs. 3e, 3f, respectively. The respective pure-dephasing times τ_2^* can be obtained via the decay constant of an exponential fit to the dephasing function. As shown in Extended Data Fig. 3g, τ_2^* strongly decreases from about 180 fs at 3 K to about 19 fs at 150 K. Such phonon-induced dephasing leads to both PL line broadening (as studied previously in Ref.27) and the elongation of the radiative lifetime (as studied in this work). Taking into account the larger QD sizes studied in the experiments (typically larger than 10 nm) as well as the known acceleration with decreasing QD size (as result of the stronger exciton-phonon coupling in smaller QDs), the

magnitude of our computationally found dephasing times for a 2.4 nm QD are also in qualitatively agreement with experiments.

Until now, we have performed the atomistic analysis of wavefunction delocalization and dephasing for the HOMO wavefunction which corresponds to the hole wavefunction of the exciton. Obtaining a complete picture of the photo physics of the excitonic state requires additionally considering the LUMO wavefunction (electron wavefunction). However, it is well known that perovskite QD models without full surface passivation create surface-localized LUMOs due to undercoordinated bromine atoms, see Extended Data Fig. 4a as well as previous works on the topic[38,39].

Fortunately, we can emulate the electrostatic environment provided by surface ligands employed in experiments via a recently developed core/shell model[27] consisting of a 2.4 nm $CsPbBr_3$ core covered by an epitaxial single layer of a wide bandgap (here: $CsCaBr_3$) material, see Extended Data Fig. 4b. Similar to the typically employed organic ligands, our wide-bandgap shell passivates the $CsPbBr_3$ core and thus enables delocalization of the LUMO wavefunction in the core (Extended Data Fig. 4c left) without effects on the HOMO wavefunction of the core (Extended Data Fig. 4c right). With such a surface passivation model, we can now also target the notoriously difficult LUMO (de-)localization.

Extended Data Fig. 4d displays snapshots of the density distribution of the LUMO wavefunction projected onto the y-z plane from AIMD trajectories obtained at 3 K (left image) and 150 K (right image). The time-averaged IPR of the LUMO is displayed in Extended Data Fig. 4e showing reduced FWHM at higher temperature. In addition, time-averaged RMSD of the center of the LUMO density from the QD center shows increasing variations in the position of the

wavefunction (Extended Data Fig. 4f). As a further support, we included a movie of the dynamics of the wavefunctions during the AIMD simulations as other supplementary materials.

Employing the core/shell QD model, in Extended Data Figs. 4g-i, we present the dephasing analysis for the LUMO, at both 3 K and 150 K. Similar as for the HOMO, the pure-dephasing time τ_2^* strongly decreases with increasing temperature, from above 80 fs at 3 K to below 20 fs at 150 K. Finally, availability of both LUMO and HOMO also allows us to retrieve the dephasing of the energy gap, *i.e.,* the quantity relevant for photon emission/absorption. Extended Data Figs. 4j-l show that also here interaction with phonons at elevated temperature reduces the pure-dephasing time τ_2^* markedly, from about 150 fs at 3 K to below 20 fs at 150 K.

In summary, the dephasing analysis of the HOMO in the core-only QD as well as the dephasing analysis of the HOMO, LUMO, and energy gap in the core/shell QD all conclude the same qualitative trend: interaction with phonons is a major source for pure dephasing, accelerating by about one order of magnitude when increasing the temperature from 3 K to 150 K.

Finally, to check for the robustness of our findings of phonon-induced dephasing presented above in Extended Data Figs. 3 and 4, we further compared the obtained pure-dephasing times for different AIMD ensembles (the canonical ensemble NVT and the micro-canonical ensemble NVE) and for different atomistic QD models (a 2.4 nm $CsPbBr_3$ core-only QD and a 2.4/3.6 nm $CsPbBr_3/CsCaBr_3$ core/shell QD). As shown in Fig. S5, the clear trend of phonon-induced acceleration of dephasing with increasing temperature is robust against all these variations: while for the core-only QD at 3 K we note slightly (2-3 times) faster dephasing with the NVT ensemble (first column) than with the NVE ensemble (second column), likely due to the finite thermostat action in the former, the difference reduced to a mere 10% at 150 K and, importantly, is always much smaller than the 1-2 orders of magnitude phonon-induced acceleration upon increasing the

temperature from 3 K to 150 K. Similar minor variations are found when comparing core-only QD (first column) and core/shell QD (third column). In conclusion, we have presented robust computational evidence of phonon-induced exciton wavefunction localization and acceleration of dephasing in $CsPbBr_3$ QDs, in line with our experimental findings of temperature-induced prolongation of the radiative lifetime (Fig. 3). Moreover, these new simulations reveal unique atomistic insights into how the activation of phonons at higher temperatures induces wavefunction localization, and, hence, a loss of superradiance via the thermally induced collapse of the giant coherent volume of excitons. Overall, our finding of thermally activated electronic wavefunction localization in perovskite QDs constitutes a solid-state analogue of a well-established process in molecular J-aggregates. In the latter, phonons localize the electronic wavefunction on a single monomer with a resulting net increase in radiative lifetime at higher temperatures[40].

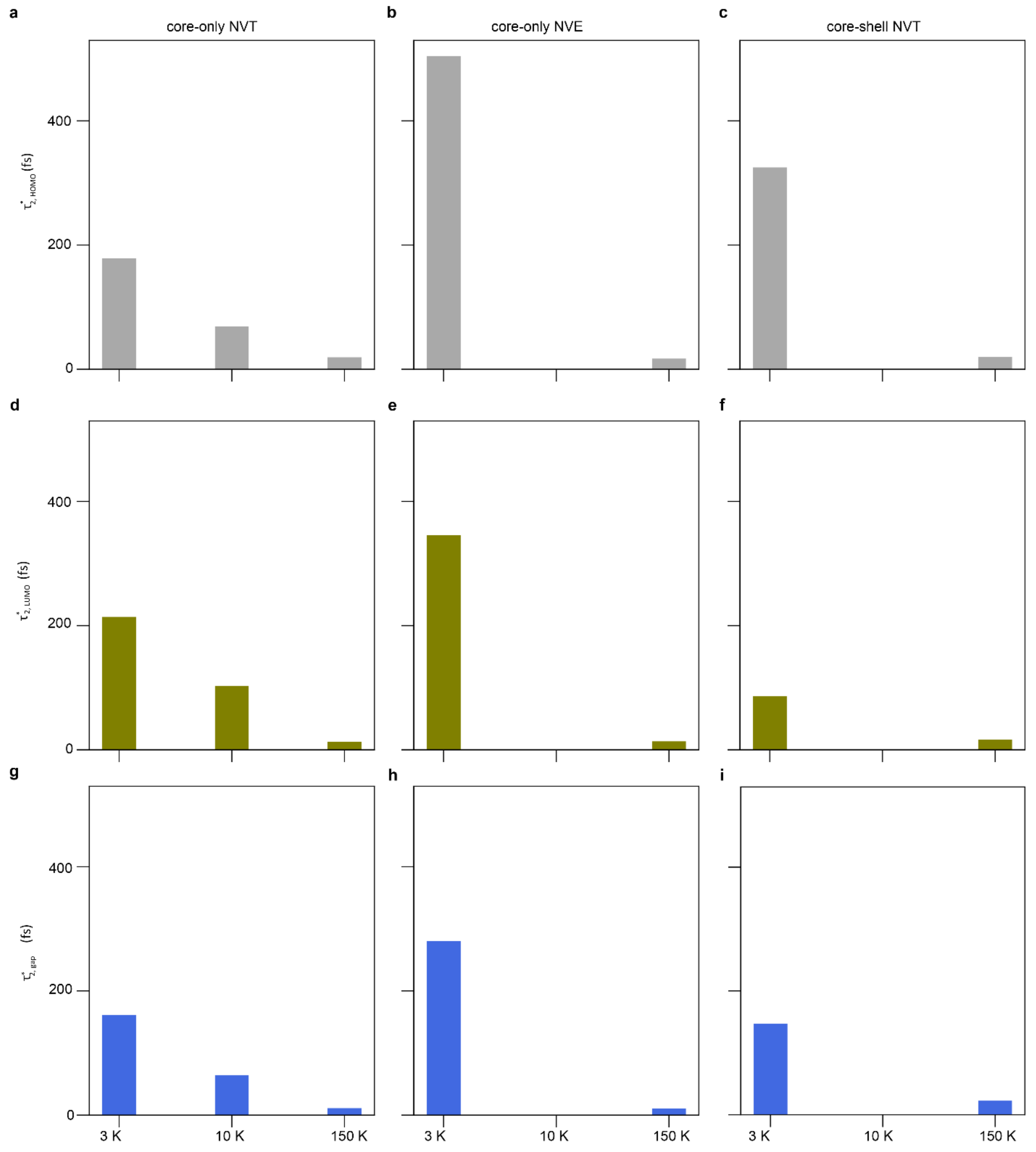

Fig. S5 | Robust finding of phonon-induced dephasing across different atomistic QD models and AIMD ensembles. A strong acceleration of pure dephasing with increasing temperature is found for HOMO (a-c), LUMO (d-f), and energy gap (g-i), with a qualitative trend independent of the employed AIMD ensemble (NVT in a,d,g vs. NVE in b,e,h) or atomistic model (2.4 nm $CsPbBr_3$ core-only QD in first column vs. 2.4/3.6 nm $CsPbBr_3/CsCaBr_3$ core/shell QD in third column).

IV. Photon statistics of exciton and biexciton emission

For the $g^{(2)}$ studies, QDs are excited under higher fluence around 0.85 μJ/cm^2 to obtain sufficient signal-to-noise ratio for the APDs. Under these conditions, we observe biexciton emission with an intensity of 20-30 % of the exciton intensity, as the spectra (red curve) shown in Fig. 5a.

The relative intensity of biexciton emission compared to exciton emission depends on the number of excitations per pulse (<N>) as well as the photoluminescence quantum yield (QY) of exciton and biexciton. <N> is proportional to fluence, intrinsic absorption coefficient of the semiconductor material and the QD volume. For large $CsPbBr_3$ QDs, <N> is difficult to assess since literature values for the intrinsic absorption cross section span an entire order of magnitude[41,42] and due to difficulties in determining the exact volume of a large QD under investigation. The latter is a result of the fact that PL energy is near size-independent for QDs >20 nm preventing a reliable size assignment of single QDs based on their PL spectra. In addition, small changes in the particle size can result in considerable changes in the volume and hence absorption cross section. Without a reliable prediction of <N>, it is not possible to determine the relative intensity of the biexciton from the spectrum which in turn limits our knowledge of the biexciton QY. However, the absence of anti-bunching in the $g^{(2)}$ suggests that multi-excitons exhibit QYs that are very similar to the exciton QY. Owing to the size-dependence of the biexciton QY in QDs[43-45], identical exciton and biexciton QYs have previously been reported in perovskite QDs with edge lengths >20 nm[46].

To investigate if anti-bunching in $g^{(2)}$ can be weak or absent despite low biexciton intensity (e.g. Fig 5a and d), we performed Monte Carlo simulations of (multi-)exciton emission. Based on the discussion above, we assume that the exciton, biexciton and multiexcitons feature similarly

high QYs and thus we set the QY of all to 1. We can then investigated the effect of <N> on biexciton to exciton intensity ratio and on $g^{(2)}$. In Extended Data Fig. 8a, we display the relative intensity of exciton and biexciton for <N>=0.1. Despite the negligible biexciton emission, the $g^{(2)}$ is not anti-bunched (Extended Data Fig. 8b), attesting near-Poissonian photon statistics. We next performed the same simulations for <N>=1 to better represent our $g^{(2)}$ measurements at high flux and obtained the results displayed in Extended Data Fig. 8c and d. The relative intensities of exciton and biexciton now resemble our experimental spectra and we do not observe anti-bunching. Note that exclusion of noise and losses would further weaken the anti-bunching. On the contrary, if the QYs of multiexciton are extremely low, single QD should behave as a perfect single photon emitter. Simulation for <N>=1 and 0% QYs of multiexciton (Extended Data Fig. 8e and f) show perfect anti-bunching for single QD, confirming that filtering out the red-shifted biexciton emission can transform the $g^{(2)}$ from not anti-bunched to anti-bunched (Extended Data Fig. 8d and f).

References

1 Krieg, F. *et al.* Colloidal $CsPbX_3$ (X= Cl, Br, I) nanocrystals 2.0: Zwitterionic capping ligands for improved durability and stability. *ACS Energy Lett.* **3**, 641-646 (2018).

2 Bera, S., Behera, R. K. & Pradhan, N. α-halo ketone for polyhedral perovskite nanocrystals: Evolutions, shape conversions, ligand chemistry, and self-assembly. *J. Am. Chem. Soc.* **142**, 20865-20874 (2020).

3 Becker, M. A. *et al.* Bright triplet excitons in caesium lead halide perovskites. *Nature* **553**, 189-193 (2018).

4 Shcherbakov-Wu, W., Sercel, P. C., Krieg, F., Kovalenko, M. V. & Tisdale, W. A. Temperature-independent dielectric constant in $CsPbBr_3$ nanocrystals revealed by linear absorption spectroscopy. *J. Phys. Chem. Lett.* **12**, 8088-8095 (2021).

5 Sercel, P. C. *et al.* Exciton fine structure in perovskite nanocrystals. *Nano Lett.* **19**, 4068-4077 (2019).

6 Sercel, P. C., Lyons, J. L., Bernstein, N. & Efros, A. L. Quasicubic model for metal halide perovskite nanocrystals. *J. Chem. Phys.* **151**, 234106 (2019).

7 Krieg, F. *et al.* Monodisperse long-chain sulfobetaine-capped $CsPbBr_3$ nanocrystals and their superfluorescent assemblies. *ACS Cent. Sci.* **7**, 135-144 (2020).

8 Zhao, Q. *et al.* Size-dependent lattice structure and confinement properties in $CsPbI_3$ perovskite nanocrystals: negative surface energy for stabilization. *ACS Energy Lett.* **5**, 238-247 (2019).

9 Yang, Z. *et al.* Impact of the halide cage on the electronic properties of fully inorganic cesium lead halide perovskites. *ACS Energy Lett.* **2**, 1621-1627 (2017).

10 Blundell, S. & Guet, C. All-order correlation of single excitons in nanocrystals using a k· p envelope-function approach: Application to lead halide perovskites. *Phys. Rev. B* **105**, 155420 (2022).

11 Kane, E. O. Band structure of indium antimonide. *J Phys Chem Solids* **1**, 249-261 (1957).

12 Sercel, P. C. in *HYBRID ORGANIC INORGANIC PEROVSKITES: Physical Properties and Applications: Volume 1-Hybrid Organic Inorganic Perovskites: Physical Properties* 63-113 (2022).

13 Hanamura, E. Very large optical nonlinearity of semiconductor microcrystallites. *Phys. Rev. B* **37**, 1273 (1988).

14 Hanamura, E. Rapid radiative decay and enhanced optical nonlinearity of excitons in a quantum well. *Phys. Rev. B* **38**, 1228 (1988).

15 Rashba, E. & Gurgenishvili, G. Edge absorption theory in semiconductors. *Sov. Phys. Solid State* 4, 759–760 (1962).

16 Miyata, K. *et al.* Large polarons in lead halide perovskites. *Sci. Adv.* **3**, e1701217 (2017).

17 Sultanova, N. G., Nikolov, I. D. & Ivanov, C. D. Measuring the refractometric characteristics of optical plastics. *Opt Quantum Electron* **35**, 21-34 (2003).

18 Nguyen, T., Blundell, S. & Guet, C. Calculation of the biexciton shift in nanocrystals of inorganic perovskites. *Phys. Rev. B* **101**, 125424 (2020).

19 Gramlich, M., Swift, M W., Lampe, C., Lyons, J. L., Doblinger, M., Efros, Al. L., Sercel, P C., Urban, A. S., Dark and Bright Excitons in Halide Perovskite Nanoplatelets, Adv. Sci. **9**, 2103013 (2021).

20 Efros, A. L. & Rosen, M. The electronic structure of semiconductor nanocrystals. *Annu. Rev. Mater. Sci.* **30**, 475-521 (2000).

21 Grigoryev, P. S., Belykh, V. V., Yakovlev, D. R., Lhuillier, E. & Bayer, M. Coherent spin dynamics of electrons and holes in $CsPbBr_3$ colloidal nanocrystals. *Nano Lett.* **21**, 8481-8487 (2021).
22 Huynh, U. N. *et al.* Transient quantum beatings of trions in hybrid organic tri-iodine perovskite single crystal. *Nat. Commun.* **13**, 1428 (2022).
23 Ramade, J. *et al.* Fine structure of excitons and electron–hole exchange energy in polymorphic $CsPbBr_3$ single nanocrystals. *Nanoscale* **10**, 6393-6401 (2018).
24 Dicke, R. H. Coherence in spontaneous radiation processes. *Phys. Rev.* **93**, 99 (1954).
25 Tighineanu, P. *et al.* Single-photon superradiance from a quantum dot. *Phys. Rev. Lett.* **116**, 163604 (2016).
26 Scully, M. O. Collective Lamb shift in single photon Dicke superradiance. *Phys. Rev. Lett.* **102**, 143601 (2009).
27 Rainò, G. *et al.* Ultra-narrow room-temperature emission from single $CsPbBr_3$ perovskite quantum dots. *Nat. Commun.* **13**, 2587 (2022).
28 Yazdani, N., Volk, S., Yarema, O., Yarema, M. & Wood, V. Size, ligand, and defect-dependent electron–phonon coupling in chalcogenide and perovskite nanocrystals and its impact on luminescence line widths. *ACS Photonics* **7**, 1088-1095 (2020).
29 Kühne, T. D. *et al.* CP2K: An electronic structure and molecular dynamics software package-Quickstep: Efficient and accurate electronic structure calculations. *J. Chem. Phys.* **152**, 194103 (2020).
30 VandeVondele, J. *et al.* Quickstep: Fast and accurate density functional calculations using a mixed Gaussian and plane waves approach. *Comput. Phys. Commun.* **167**, 103-128 (2005).
31 Goedecker, S., Teter, M. & Hutter, J. Separable dual-space Gaussian pseudopotentials. *Phys. Rev. B* **54**, 1703 (1996).
32 Hartwigsen, C., Gœdecker, S. & Hutter, J. Relativistic separable dual-space Gaussian pseudopotentials from H to Rn. *Phys. Rev. B* **58**, 3641 (1998).
33 Krack, M. Pseudopotentials for H to Kr optimized for gradient-corrected exchange-correlation functionals. *Theor. Chem. Acc.* **114**, 145-152 (2005).
34 Perdew, J. P., Burke, K. & Ernzerhof, M. Generalized gradient approximation made simple. *Phys. Rev. Lett.* **77**, 3865 (1996).
35 Bussi, G., Donadio, D. & Parrinello, M. Canonical sampling through velocity rescaling. *J. Chem. Phys.* **126**, 014101 (2007).
36 Shi, R. *et al.* Structural Disorder in Higher-Temperature Phases Increases Charge Carrier Lifetimes in Metal Halide Perovskites. *J. Am. Chem. Soc.* **144**, 19137-19149 (2022).
37 Mukamel, S. *Principles of nonlinear optical spectroscopy*. (Oxford University Press, USA, 1995).
38 Bodnarchuk, M. I. *et al.* Rationalizing and controlling the surface structure and electronic passivation of cesium lead halide nanocrystals. *ACS Energy Lett.* **4**, 63-74 (2018).
39 du Fossé, I. *et al.* Limits of Defect Tolerance in Perovskite Nanocrystals: Effect of Local Electrostatic Potential on Trap States. *J. Am. Chem. Soc.* **144**, 11059-11063 (2022).
40 Potma, E. O. & Wiersma, D. A. Exciton superradiance in aggregates: The effect of disorder, higher order exciton-phonon coupling and dimensionality. *J. Chem. Phys.* **108**, 4894-4903 (1998).
41 Maes, J. *et al.* Light absorption coefficient of CsPbBr3 perovskite nanocrystals. *J. Phys. Chem. Lett.* **9**, 3093-3097 (2018).

42 Makarov, N. S. *et al.* Spectral and dynamical properties of single excitons, biexcitons, and trions in cesium–lead-halide perovskite quantum dots. *Nano Lett.* **16**, 2349-2362 (2016).
43 Nair, G., Zhao, J. & Bawendi, M. G. Biexciton quantum yield of single semiconductor nanocrystals from photon statistics. *Nano Lett.* **11**, 1136-1140 (2011).
44 Robel, I., Gresback, R., Kortshagen, U., Schaller, R. D. & Klimov, V. I. Universal size-dependent trend in auger recombination in direct-gap and indirect-gap semiconductor nanocrystals. *Phys. Rev. Lett.* **102**, 177404 (2009).
45 Li, Y., Luo, X., Ding, T., Lu, X. & Wu, K. Size - and Halide - Dependent Auger Recombination in Lead Halide Perovskite Nanocrystals. *Angew. Chem.* **132**, 14398-14401 (2020).
46 Igarashi, H., Yamauchi, M. & Masuo, S. Correlation between single-photon emission and size of cesium lead bromide perovskite nanocrystals. *J. Phys. Chem. Lett.* **14**, 2441-2447 (2023).
